# Fiber-Optic Chemical Sensors and Fiber-Optic Bio-Sensors

**DOI:** 10.3390/s151025208

**Published:** 2015-09-30

**Authors:** Marie Pospíšilová, Gabriela Kuncová, Josef Trögl

**Affiliations:** 1 Czech Technical University, Faculty of Biomedical Engeneering, Nám. Sítná 3105, 27201 Kladno, Czech Republic; E-Mail: pospim14@fbmi.cvut.cz; 2 Institute of Chemical Process Fundamentals, ASCR, Rozvojová 135, 16500 Prague, Czech Republic; E-Mail: kuncova@icpf.cas.cz; 3 Faculty of Environment, Jan Evangelista Purkyně University in Ústí nad Labem, Králova Výšina 3132/7, 40096 Ústí nad Labem, Czech Republic

**Keywords:** fiber-optic sensor, chemical sensor, enzymatic sensor, whole cell biosensor, immobilization of biologicals, tapered optical fiber

## Abstract

This review summarizes principles and current stage of development of fiber-optic chemical sensors (FOCS) and biosensors (FOBS). Fiber optic sensor (FOS) systems use the ability of optical fibers (OF) to guide the light in the spectral range from ultraviolet (UV) (180 nm) up to middle infrared (IR) (10 µm) and modulation of guided light by the parameters of the surrounding environment of the OF core. The introduction of OF in the sensor systems has brought advantages such as measurement in flammable and explosive environments, immunity to electrical noises, miniaturization, geometrical flexibility, measurement of small sample volumes, remote sensing in inaccessible sites or harsh environments and multi-sensing. The review comprises briefly the theory of OF elaborated for sensors, techniques of fabrications and analytical results reached with fiber-optic chemical and biological sensors.

## 1. Introduction

The progress in photonics, information technology and biotechnology has involved an investigation of new sensor systems employing optical fibers. Fiber optic sensor (FOS) systems use the ability of optical fibers (OF) to guide the light in the spectral range from UV (180 nm) up to middle IR (10 µm) (depending on the material of the core of the OF). Extrinsic FOS use OF only as light waveguides. In case of intrinsic FOS, parameters of guided light are more or less altered by the physical-optical, chemical or biological parameters of the surrounding environment of the core of the OF. Those parameters can include the refractive index, concentration of chemical or biological elements, pH, pressure and temperature.

The introduction of OF in the sensor system has brought a number of advantages such as miniaturization of the device, geometrical flexibility, measurement of small sample volumes, remote sensing in normally inaccessible sites or harsh environments, multi-sensing possibility, continuous quantitative or qualitative measurement and the immunity of an OF to electrical noises. Chemical and thermal stability of quartz glass, which is the material of prevailing OF for the spectral range from ultraviolet to mid infrared, are comparable only with platinum.

The development and investigation of fiber optic chemical sensors (FOCS) and biosensors (FOBS) has been reflected by more than thousands references devoted to this field in 2002–2015 reviewed in [1,2,3,4,5,6,7,8,9]. The fundamental principles of FOS operation were described [10,11,12,13,14,15] and the latest developments in optical fiber devices and their applications to sensor technology in various areas of industry, transportation, communication, security and defense, as well as daily life, are presented in [16].

Chemists used to employ sensors with electrodes as pH, Clark and ion-selective electrode. Among fiber optical chemical sensors, which are less commercially available, only sensors of oxygen become more used. This review is addressed to analytical chemists and chemical engineers who think of a substitution of standard laboratory analyses with FOCS or FOBS. The review starts with an overview of the optical fibers, which were developed for optical sensors and the mathematical description of the electromagnetic field (EM) distribution in OF as a function of OF geometry and profile of refraction index. The mathematical relations give a qualitative assessment of FOS in the reflection and transmission arrangements, and light propagation in U-shaped and tapered up and down fibers. Overview of FOCS is enriched with original results of pH measurements with tapered FOS in small volume samples.

The range of biosensor designs and applications is tremendous and therefore we focus on enzyme and whole-cell optical fiber biosensors, both in the group of catalytic sensors, applicable in clinical medicine, biotechnologies and monitoring pollution. The subdivisions deal with the narrow field of enzymatic sensors with optical oxygen transducers and whole cell sensors for monitoring environmental pollution.

## 2. Optical Principles of Fiber Chemical Sensors and Biosensors

### 2.1. Optical Fibers for Chemical Sensors and Biosensors

**Figure 1:**
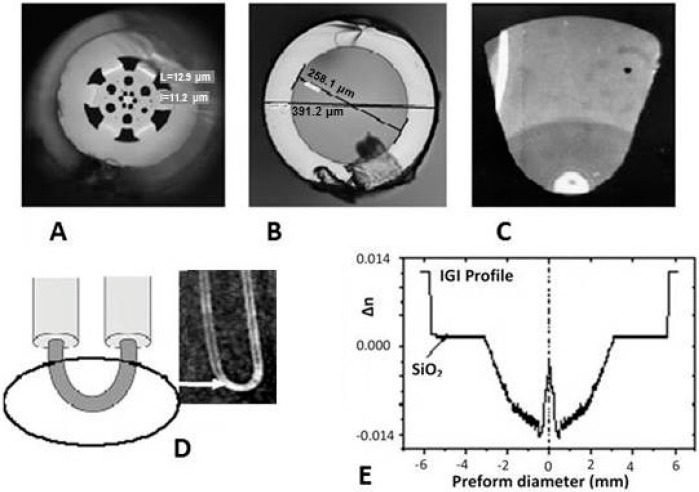
Optical fibers for sensor applications—(**A**) a photo of a Polymer clad microstructure fiber; (**B**) a photo of a hollow fiber; (**C**) a photo of a part of S-Fiber; (**D**) a scheme of the U-fiber and a photo of the real U-shape part of a probe (reproduced from [46]); (**E**) Refractive index profile—inverted gradient index (IGI) in preform fabricated by the Modified chemical vapor deposition method at Institute of Photonics and Electronics (IPE) Prague; the photo (E) reproduced from [47] with the permission of the Czech Technical University (CTU) Editors.

**Figure 2:**
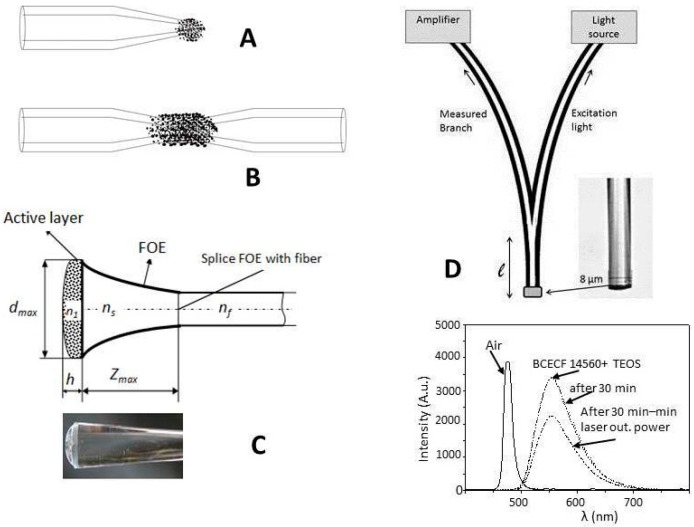
Tapered fiber (**A**) One conus with active layer on a tip; (**B**) Two conus with active layer; (**C**) Tapered up fiber optic element (FOE) with active layer and a photo of FOE with microorganisms immobilized in silica gel; (**D**) Schema of V-taper with a photo of a real tip V-taper and a graph of the dependence of measured fluorescence on the wavelength in the measure branch of V-taper. Strength line—without active layer (no fluorescence) and dash line—with active layer on the tip for two different output powers of laser and time after coating of layer.

The majority of applications of FOS in biology and medicine operate in the visible and near infrared spectral range (from 340 nm up to 2 µm). Suitable types of fibers for this spectral range are fibers based mainly on SiO_2_. They are Single-Mode—SM, Multi-Mode—MM with Step Index—SI such as PCS (Polymer Cladding Silica) fibers or Gradient Index (GI) or fiber bundles made from those fibers [17]. Specially designed fibers of the same material include microstructure fibers or PCF fibers (Polymer clad Microstructure Fiber), hollow fibers (capillary), sectorial fibers (S-fibers), U-shaped fibers, fibers with inverted gradient index profile (IGI, see Figure 1A–E) and down or up tapered fibers (see Figure 2A,C) [18,19,20,21,22,23]. The types of fibers mentioned above are now produced commercially [24,25]. The development and investigation of designed OF in that way has been done with the aim of increasing the evanescence field [26,27,28,29,30] resulting in increasing the sensitivity and detection limit of FOS. One way was two-cone tapered fiber (see Figure 2B) used to determine the concentration of chemicals surrounding the tapered OF in the transmission arrangement [1,2,3] as well as the development of tapered fiber optical elements (FOE, see Figure 2C,D). The new type of FOE the called V-taper (see Figure 2D), was developed for FOCS in the reflection arrangement for measurement in human, animal, plant cells or very small volumes (order µL) of special liquids (urea, a drop of perspiration, and plant exudates). The polymer fibers (POF) for the visible spectra range are often used in industry, biology and medicine due to their low cost and mechanical properties [18,30,31,32,33]. This type of fibers compared with silica base fibers is more flexible and cheaper. Their numeric aperture (NA) (0.4–0.5) is higher than silica-based fiber (0.1–0.28) and the attenuation of POF is around of 1 dB/m at 650 nm. The sensor system using the microstructure polymer optical fiber was described in [31]. Chalcogenide fibers were developed for middle IR spectral range up to 10.6 µm—the wavelength of a CO_2_ laser [8,32,33]. The application of chalcogenide fibers into FOCS or FOBS in the medical field is limited by their disadvantages such as toxic and fragile fiber materials. That disadvantages have been overcome by the development of new design of hollow fibers (silica or glass capillary) with an internal total reflection metal layer (Au, Ag) determined for a spectral range of wavelength >2.5 µm [34,35]. A comprehensive overview of the fabrication, development and types of OF is to be found for example in [17]. The new FOE—OF with a long period grating (FLPG) or Bragg grating FBG—was developed for fiber optic sensors (FOS) to increase the sensitivity of the sensor system on the base of interference principle. The authors described the technology of FLPG fabrication and its application in the sensor system [36,37,38,39,40].

Optical micro-resonators are new FOE using the so called “Whispering-gallery-mode” (WGM) can be described by circulating electromagnetic waves which are strongly confined within a structure. Their great potential for chemical and biological sensor applications has been intensively investigated over the last ten years [41,42]. Optical bottle micro-resonators have been fabricated from short sections of optical fiber by the focused beam of a CO_2_ laser. Microspheres with diameters around 330 µm were prepared by melting a tip of silica fiber into a micro-ball and subsequently were modified by a thin silica xerogel layer using tetraethoxysilane-based sol. Shifts of resonance dips caused by acetone vapors have been observed [43,44]. A bio-sensing application of WGM by using a silica microsphere near 100 µm of radius is described in [45].

FOS systems intended for applications in biology and medicine utilize the principles mainly of optical spectroscopy in conjunction with a chemical or biological system. They can be generally divided into two basic groups:

(1) A chemical sensor defined by Wolfbeis [3] as “miniaturized analytical devices that can deliver real-time and on-line information on the presence of specific compounds or ions in complex samples” using OF (FOCS) [1,2,3]

(2) A biosensor is defined as an analytical device, which converts the concentration of the target substance into an electrical signal through a combination of a biological recognition system associated with a physical-chemical transducer and using OF (FOBS) [10].

Note: A special group of optical sensors are photonics sensors using the Raman Effect (Raman spectroscopy), computed, coherent tomography (CT) for a noninvasive medical method. An examination of tissue or other biological or chemistry material using optical fibers is also a theme for a new investigation and application of optical fiber. The high frequency transmission of a standard MM OF is a range of MHz. This fact and the reflectivity of some biology material (epithelial for example) were studied by a laboratory developed system based on laser reflectometry using optical fibers [48,49].

### 2.2. Light Propagation in Optical Fibers and Optical Fiber Elements

The physical principles of the interaction of light with mass (interrogation characteristics) in FOS are used in two ways: direct and indirect detections. The suitable dyes—transducer or other chemical or biological material are used in the case of indirect detection. The fundamental optical phenomena are attenuation α(λ), transmission *T*(λ), reflection *R*(λ), fluorescence *I*_F_(λ), phosphorescence *I*_P_(λ), bioluminiscence *I*_B_(λ), they are used in FOS systems to monitor a wide range of chemical or biology quantities.

The light—electromagnetic field (EM), which is described by the vectors of the electric field $\vec{E}$ and magnetic field $\vec{B}$—is guided by optical fiber with a radius a and refractive index profile *n*_1_(*r*) of the fiber core and refractive index *n*_2_ of the cladding if two conditions are satisfied:

(1) *n(r)* > *n*_2_,
(2) *2*π*n*_2_ < β < 2π*n*_1_; β is a propagation constant.

EM field distribution, in the core of optical fiber for the step (SI) and gradient (GI) refractive index profile (n(r)), was received in the analytical form as the result of a solution of Maxwell equations (MR). The analytical forms for $\vec{E}$ and $\vec{B}$ were used to compute the power distribution of guidance light in core *P*_0_ and non-absorbing cladding *P*_c_ of OF as is expressed in Equation (1) in the approach of wave optics [48,49,50]:

(1) $$\begin{array}{r} \frac{P_{c}}{P}=1-\frac{P_{0}}{P}\\ \frac{P_{c}}{P}={\left[\frac{u_{m}^{\infty }a}{V}\right]}^{4}\left(1-\frac{2}{V}\right) \end{array}$$

where: *V* is a normalized frequency defined as

*V = (2*π*a/*λ)∙*NA* (1a)

$u_{m}^{\infty }$ is a root of the Bessel function; *m* = 0, 1, 2, … $NA=\sqrt{\left[n_{1}{\left(r\right)}^{2}-{n_{2}}^{2}\right]}$—Numeric Aperture of OF.

Equation (1) shows the alterations of the power distribution of light between a core and cladding as the function of OF parameters (geometry, refraction index profile and refraction index value).

The most FOS systems use the principle of reflection or transmission of light when the sensing chemistries are immobilized in a layer on the tip or cladding of the OF. In this case the absorption coefficient *α_a_* of that material has to be considered. The following section provides the basic relations for the reflection coefficients on the core/cladding interface or on the tip of the OF. They were derived in the approach of RO, which is valid for the case of MM OF types and the mode can be connected with the direction of ray propagation. The absorption of the cladding material was considered via its *α_a_* [51,52]. In general, the refractive index *n* is a complex number expressed as

(2) $$n=n_{r}+in_{i}$$

The real part *n_r_* is known from Snell’s Law, the imaginary part *n_i_* is connected with *α_a_*, (usually it is used in absorption spectroscopy) by the relation

(3) $$n_{i}=\frac{{\text{α}}_{a}\text{λ}}{4\text{π}}$$

where λ is wavelength of light in vacuum.

Preferably, for some applications the refractive index *n* expressed by material constants ε*_m_* and μ*_m_* (generally complex numbers) is used:

(4) $$n={\sqrt{{\text{ε}}_{m}\text{μ}}}_{m}$$

The reflection coefficient R for ray’s incident on that interface defined by n determines a part of the back reflected power P_R_. In the case of OF, R is connected with the refraction indexes of the core and cladding. The reciprocal relation between R and P_R_ is expressed by:

(5) $$P_{R}={\sum_{k}P_{k}=\sum_{k}P_{0k}\left|R\right|}^{2N}$$

where: *k* = 1, 2,…….*V*^2^/2 is a number of guided modes.

*N* is the number of the reflection of light on the interface core/cladding and it is a linear function of the path length *L*.

If the reflection of the ray on the layer forms with numbers of *N_L_*, the different layers from dielectric materials (ε_j_) of a thickness d_j_, the reflection coefficients for TE and TM polarization are given by Equation (6):

(6) $$R^{\left(TE\right)}={\left|r^{\left(TE\right)}\right|}^{2}={\left|\frac{-MS_{21}-q_{0}q_{N+1}MS_{12}+i\left(q_{0}MS_{11}-q_{N+1}MS_{22}\right)}{+MS_{21}-q_{0}q_{N+1}MS_{12}+i\left(q_{0}MS_{11}+q_{N+1}MS_{22}\right)}\right|}^{2}\;R^{\left(TM\right)}={\left|r^{\left(TM\right)}\right|}^{2}={\left|\frac{-MP_{21}-q_{0}q_{N+1}\frac{MP_{12}}{{\text{ε}}_{0}{\text{ε}}_{N+1}}+i\left(q_{0}\frac{MP_{11}}{\varepsilon_{0}}-q_{N+1}\frac{MP_{22}}{{\text{ε}}_{N+1}}\right)}{+MP_{21}-q_{0}q_{N+1}\frac{MP_{12}}{{\text{ε}}_{0}{\text{ε}}_{N+1}}+i\left(q_{0}\frac{MP_{11}}{{\text{ε}}_{0}}+q_{N+1}\frac{MP_{22}}{{\text{ε}}_{N+1}}\right)}\right|}^{2}$$

The components in Equation (6) are defined by relation Equation (7):

(7) $$MS=\prod_{j=1}^{N+1}\left(\begin{array}{cc} \cos\left(\arg_{j}\right) & -\frac{\sin\left(\arg_{j}\right)}{q_{j}}\\ q_{j}\sin\left(\arg_{j}\right) & \cos\left(\arg_{j}\right) \end{array}\right)\;MP=\prod_{j=1}^{N}\left(\begin{array}{cc} \cos\left(\arg_{j}\right) & -{\text{ε}}_{j}\frac{\sin\left(\arg_{j}\right)}{q_{j}}\\ \frac{q_{j}}{\varepsilon_{j}}\sin\left(\arg_{j}\right) & \cos\left(\arg_{j}\right) \end{array}\right)$$

where $q_{j}=\sqrt{{\text{ε}}_{j}-{\text{ε}}_{0}\sin^{2}\text{ψ}}\;\arg_{j}=\frac{2\text{π}}{\text{λ}}q_{j}d_{j}$.

*MS_ij_* and *MP_ij_* are the matrix elements of *MS or MP* respectively; *j* is the number of layer; ε_0_ is the electrical permittivity of the core material; ε_N+1_ corresponds to the refractive index of the surrounding environment of the layers.

Note: nonmagnetic material has μ*_m_* = μ_0_ = 1 magnetic permeability of vacuum.

The total refractive index *R* is then defined for unpolarized light as:

(7a) $$\begin{array}{cc} R=\frac{1}{2}\left(R^{\left(TE\right)}+R^{\left(TM\right)}\right) & \end{array}$$

**Figure 3:**
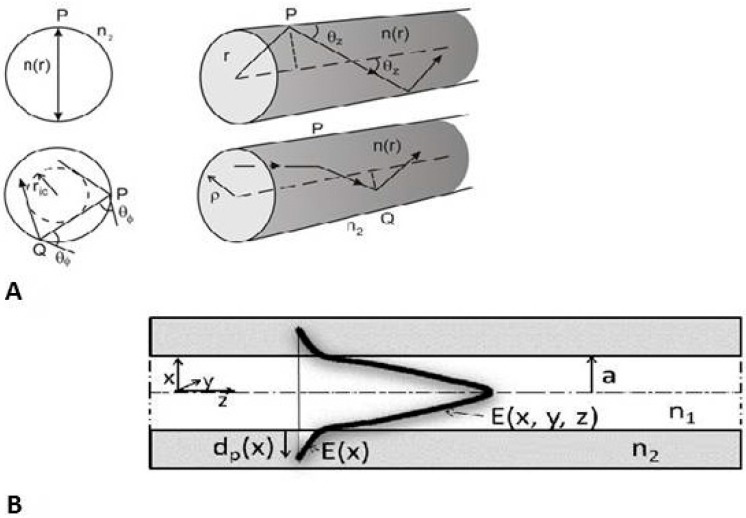
(**A**) Scheme of rays (meridian rays cross the fiber axis and scew rays do not cross the fiber axis) in the core of the optical fiber in the approach of geometric optics; (**B**) Scheme of Evanescence field *E*(*x*) and penetration depth *d_p_*(*x*). Reproduce from [47] with the permission of the CTU editors.

Relations Equations (6), (7) and (7a) are used for modeling the guided light by the OF with attenuation cladding. The analytical approximations of *P_k_* were derived for the light guide in the core fiber in the past and using the relation for *P_k_* through the Beer-Lambert law with a modified absorption coefficient γ_i_ [50,51,52]. The power *P_k_* of the ray spread by the core fiber under angle ψ with a fiber axis can be written, in cases with the absorption coefficient *α_a_*, as:

(8) $$\begin{array}{l} P_{k}=P_{0k}e^{-{\text{γ}}_{i}L}\\ {\text{γ}}_{i}\approx {\text{α}}_{a}\frac{\text{λ}n_{2}\cos\text{ψ}\cot\text{ψ}}{\text{π}an_{1}^{2}\cos^{2}{\text{ψ}}_{c}\sqrt{\cos^{2}{\text{ψ}}_{c}-\cos^{2}\text{ψ}\sin^{2}{\text{θ}}_{\varphi }}} \end{array}$$

$\psi_{c}=\arcsin\left(\frac{n_{2}}{n_{1}}\right)$ and $\psi \geq \psi_{c}$; ${\text{θ}}_{\varphi }$ is angle of the “skew” rays (see Figure 3A).

The maximum *P*_k_ is for a ray with ${\text{θ}}_{\varphi }$ = $\frac{\text{π}}{2}$. Those rays never crossed the fiber core axis.

Another approach to the *R* calculation is the model of “equivalent thickness”. This model is advantageously used for the calculation of the EM field in the fiber core which is surrounded by an attenuation layer on the cladding or on the tip of a core. This case corresponds with the fluorescence type of FOS. The optical losses were included as well by the application of the Beer—Lambert law, which described the attenuation of intensity in an absorption environment as an exponential function of its absorption coefficient and the thickness [52]. The derived relation for *R* is correct if the condition $\alpha_{a}d_{e}<<1$ is satisfied. We can then express *R* by relation Equation (9):

(9) $$R=1-\alpha_{a}d_{e};\;R^{N}=1-N\alpha_{a}d_{e}$$

Equivalent thickness is different for TE and TM polarization of light. The derived relations for *d_e_* for both polarizations are shown by relations Equation (10a,b). In the relations, *d* is the thickness of the layer on the optical cladding Equation (10a) and on the tip of the core Equation (10b):

(10a) $$\begin{array}{l} d_{e}^{\left(TE\right)}=\frac{\text{λ}n_{1}^{3}n_{2}\cos\text{ψ}}{\text{π}\left(n_{1}^{2}-n_{2}^{2}\right)\sqrt{\left(n_{1}^{2}\sin^{2}\text{ψ}-n_{2}^{2}\right)}}\\ d_{e}^{\left(TM\right)}=\frac{\text{λ}n_{1}^{3}n_{2}\cos\text{ψ}\left(2n_{1}^{2}\sin^{2}\text{ψ}-n_{2}^{2}\right)}{\pi \left(n_{1}^{2}-n_{2}^{2}\right)\sqrt{\left(n_{1}^{2}\sin^{2}\text{ψ}-n_{2}^{2}\right)}\left(\left(n_{1}^{2}-n_{2}^{2}\right)\sin^{2}\text{ψ}-n_{2}^{2}\right)} \end{array}$$

(10b) $$\begin{array}{l} d_{e}^{\left(TE\right)}=\frac{4dn_{1}n_{2}\cos\psi }{\left(n_{1}^{2}-n_{3}^{2}\right)}\\ d_{e}^{\left(TM\right)}=\frac{4dn_{1}\cos\psi \left[n_{1}^{2}\left(n_{2}^{4}+n_{3}^{4}\right)\sin^{2}\psi -n_{2}^{4}n_{3}^{2}\right]}{n_{2}^{3}\left(n_{1}^{2}-n_{3}^{2}\right)\left(\left(n_{1}^{2}+n_{2}^{2}\right)\sin^{2}\psi -n_{3}^{2}\right)} \end{array}$$

*n*_1_, *n*_2_ and *n*_3_ are refractive indexes of the fiber core, cladding and surrounding environment respectively. Relations Equations (9) and (10a,b) are valid for $\psi \geq \psi_{c}$.

Equations (6), (7), (7a) and (10b) have to be used for reflection arrangement of the FOCS, relation Equations (8), (9), (10) and (10a) can be used for modeling a transmission sensor, where a sensing layer is a part of the fiber cladding.

The bend of OF with a radius R is another way how to increase evanescence field which is used in FOS. The critical radius *R_c_* (see Equation (11)) was derived as a limit fiber bend, when the guided light could be affected by this bend. Two possibilities can occur:

(1) for each bend diameter *R > R_c_*, the guided light is not affected by *R*,

(2) for each *R* < *R_c_*, the guided light is affected by *R* due to the reduction of the number *M*(*R*) of the modes see Equations (11) and (12):

(11) $${R_{c}}^{MM}≅\frac{3{n_{1}}^{2}\text{λ}}{4\text{π}\sqrt{{\left({n_{1}}^{2}-{n_{2}}^{2}\right)}^{3}}}\;{R_{c}}^{SM}≅\frac{20\text{λ}}{\sqrt{{\left({n_{1}}^{2}-{n_{2}}^{2}\right)}^{3}}}\cdot \frac{1}{2.748-0.996\frac{\text{λ}}{{\text{λ}}_{c}}}$$

(12) $$M\left(R\right)=M_{0}\left(\frac{1-2a{n_{2}}^{2}}{R\cdot NA^{2}}\right)\to \frac{\Delta M}{M}=\frac{a{n_{2}}^{2}}{R\cdot NA^{2}}$$

where: *n*_1_—the refractive index of the OF core, *n*_2_—the refractive index of the OF cladding, *a—*the OF core radius, λ_c_—the cut off wavelength = *2*π*a·NA/*2405.

In practice, U-shaped fibers employed in FOCS for the direct detection of the refractive index *n* of a media surrounding a part of a bend core utilize this fact. The bend radius *R* < *R*_c_ caused the change of the incident angles in the bend part of the fiber. The numbers of the reflected rays at the core/cladding are increased and the evanescence field penetrates in the cladding more effectively.

The evanescence field *E*(*x*) is an important phenomenon on the core/cladding boundary (see Figure 3B) in the case of Total Internal Reflection (TIR). It is a part of the electromagnetic wave which penetrates from the core to the OF cladding. The *x*-axis is perpendicular to the direction of light propagation in the *z*-axis. The penetration depth *d_P_* was derived from the definition of *E*(*x*) as is shown in relation Equation (13):

(13) $$\begin{array}{l} E\left(x\right)=E_{o}\left(a\right)e^{-\frac{x}{d_{p}}}\\ d_{p}=\frac{\text{λ}}{2\text{π}\sqrt{n_{1}^{2}\sin^{2}\text{ψ}-n_{2}^{2}}} \end{array}$$

Ψ is each incident angle for which the condition Equation (14) has to be satisfied:

(14) $$\psi \geq \psi_{c}=\sin\frac{n_{2}}{n_{1}}$$

The maximum value of *d_p_* derived from relation Equation (13) is achieved for ψ*_c_*. They represent the tools for the calculations of suitable parameters of the OF, which are used in chemical or biological senor systems in the reflection or transmission arrangements. *E*(*x*) is the part of the electromagnetic field interacting with the surrounding environment of the OF core. The way to increase *E*(*x*) was newly designed fibers or fiber optic elements for FOS applications.

Tapered down optical fibers have a typical tip or waist diameter in the scale of dimension of µm to hundreds of nm and were developed for the evanescence sensor. They reduce the V number of a standard OF and the part of the OF with a sensing layer with a refraction index different from the refractive index of the OF cladding. When we consider the reflection or transmission arrangement of FOS and the PCS fiber with a core refractive index of fused silica 1457 and cladding 141, a fiber core diameter *a* = 200 µm and a wave length of 0.633 µm, the *V*_0_ number is 728 according to relation 1a. When a part of fiber cladding is removed and replaced with new cladding with a lower refractive index, for example alginate *n* = 1334, the *V*_1_ number is 1150. The difference between *V*_0_ and *V*_1_ goes to increase the transmission loss. If the core radius *a* tapered down to new radius *a_1_* in a part with a smaller refractive index to satisfy condition *V*_0_ = *V* is *a*_1_ = 42 µm and the losses caused by different *NA* or *V* numbers will be reduced to zero. The different shapes of tapered tips for the fiber optic biosensor were experimentally analyzed by comparing the detected fluorescence of rhodamine 6G dye immobilized on the tip of the tapered fibers. The tip shape with the maximum received fluorescence for biosensor application was selected [53].

The concentration *C* of the monitoring elements of the tested media is proportionally dependent on the attenuation *α*(λ) on wavelength λ by relation Equation (15) and can be determined by a direct measurement of *α*(λ):

(15) $$\log\frac{I_{0}(\text{λ})}{I(\text{λ})}=\text{α}(\text{λ})=\text{ε}CKl$$

where: *ε*—the molar extinction coefficient of the monitoring element (ME), *C*—the concentration of ME, *K*—the arrangement constant, *l*—the optical path length in the sample.

The fluorescence value *I_F_(λ)* of the transducers used in the indirect measurement of ions, pH, O_2_ and others. The linear function in the case of a low concentration *C* can be expressed by Equation (16):

(16) $$I_{F}(\text{λ})=K\cdot I_{0}({\text{λ}}_{e})\cdot {\text{α}}_{0}({\text{λ}}_{e})\cdot Q\cdot C_{t}\cdot l$$

where: *I*_0_(*λ_e_*)—the output intensity of excitation source on wavelength λ_e_, *α_0_*(*λ_e_*)—the absorption coefficient, *Q*—the quantum yield of transducer, *C*_t_—the concentration of transducer, *K*—the arrangement constant, *l*—the optical path length in the sample.

The value of *I*_F_(λ) depends on the composition of the matrices material, where the suitable transducer is immobilized, and on the technology of the immobilization process. The concentration of quencher reduces the value of *I*_F_(λ). Those quenchers include metal ions, solvents, and gases, such as oxygen. The feature of O_2_ as a quencher is used by the FOCS to determine its concentration. The measurement of the dependence of the fluorescence of a ruthenium complex immobilized in a suitable matrix as a function of *I*_F_(λ) provides that information. The time dependence of the fluorescence of the ruthenium complex measurement was used in the developed biosensor system for food quality assurance technology. A more detailed description of that system is presented below in Section 3.2.

For the transmission arrangement of the FOS, where the suitable transducer was immobilized in the cladding of the OF, the relation Equation (15) was experimentally demonstrated for the established fluorescence signal from cladding to core as:

(17) $${I_{F}}_{c}\propto I_{0}\alpha_{t}la{\left(\frac{r_{\max}}{a}\right)}^{2}\sin^{8}\psi_{0}^{\max}NA^{-1}$$

where *I*_0_ is the intensity of the light source, *α_c_* is the absorption coefficient of the transducer, *l* is the length of the absorption layer, *a* is the radius of the core, *NA* is the numerical aperture of the fiber, *r*_max_ is the maximal radius of the light beam on the tip of the OF and ψ_0_^max^ is the maximal launching angle from the light source to the OF.

The principle of Surface Plasmon Resonance (SPR), described for example in [5,7], is employed in the FOCS and FOBS systems for high resolution in a refractive index in the range of 10^−7^–10^−8^ RIU (Refractive Index Unit). The surface plasmon is a quantum of the electron density wave which is formed as a result of the incident light of p polarization on a metal-coated dielectric material (glass prism, optical fiber or integrated optic material) when its energy and momentum are transferred onto the surface of the metal to create a surface plasmon. The thickness of the metal coating layer is some tens of nanometers. The plasmon is propagated along the glass-metal surface and decays in glass and metal as the evanescence wave. Three methods are used to increase the photon momentum and to couple into metal: (1) the Kretschmann configuration using the total internal reflection inside a prism with an electrical permittivity of ε*_p_*; (2) diffraction at a grating and wave guiding fiber and (3) integrated optics (IO). The SP wave is excited by the EM wave with *p* polarization in a metal layer with ε*_m_*. The reflected light from the prism/metal surface is a function of the incident angle as is shown in Equation (18). There is one incident angle ψ_rez_ for which is reflected intensity zero. Its value depends on the refractive index described by help of electric permittivity ε*_s_* of the surrounding area of the metal layer which is used for the refractive index measurement. The theoretical description of SPR and practical applications of SPR in the FOCS and FOBS have been published for example in [54,55,56].

(18) $$\frac{\text{ω}}{c}\sqrt{{\text{ε}}_{p}}\sin{\text{ψ}}_{res}=\frac{\text{ω}}{c}\sqrt{\frac{{\text{ε}}_{m}{\text{ε}}_{s}}{{\text{ε}}_{m}+{\text{ε}}_{s}}}$$

Localized SPR (LSPR) and propagating SPR (PSPR) modes are used in the FOBS which are described in more detail in [8].

OF with grating in the core are used as well in the FOS. Two types of gratings are fabricated in the core: Brag (FBG) and Long period gratings (FLPG). The gratings in the core of the OF are created by periodical changes in the refractive index defining the grating period Λ_e_. This period defined a wavelength of λ_B_ = 2n_ef_Λ_e_ for which the grating has a maximum of transmission. The period of the FBG is usually in microns and that of the FLPG is longer (100–500 µm)

Nowadays, the FOCS and FOBS are used in biology and medical applications predominantly in fluorescence or bioluminescence applied systems in the reflection configuration. The principle of SPR has been combined with nanotechnology and precise detection systems based on interference and the new technique of coherence detection [57,58].

## 3. Fiber Optic Chemical Sensor (FOCS)

Arnold A. Marh defined a chemical sensor as “a device that is used to measure the concentration or the activity of a chemical species in a sample of interest. “Ideally, the device should be capable of operating in a continuous and reversible manner, directly in the sample matrix” [59]. We mention here sensor systems which employ special shape fibers, sensing material on the clad of the core or the tip of the fibers firmly connected to the OF which use the principles described in Section 2. The mentioned publications [1,2,3,7] have collected the fundamental parameters and work principle of the FOCS used to detect gases (H_2_-hydrogen, HC-hydrocarbons, H_2_O_2_, O_2_, O_3_-Ozone gas, N_2_, CO_2_, NO_2_, NO, SO_2_, gas phase NH_3_-Ammonia, Cl), ions (pH-H^+^, Ca^2+^, Cu^2+^, Co^3+^, Ni^2+^, Al^3+^, Ga^2+^, Cd^2+^, Hg^2+^, Pb^2+^, Ag^+^, Fe^3+^, Zn^2+^) and special chemical compounds for environmental measurements (pollutants, agrochemical and nerve agents, drugs and pharmaceuticals).

In the FOCS, the detection structures combining reflection and absorption was used with success employing U-shaped fibers, tapered fibers, FBG or FLPG and SPR technique which is now more focused on integrated optics (IO).

### 3.1. Components of FOCS

Since 1980 when one of the first FOS systems, called “probe” was published [60,61], a number of books and papers have been published. The “probe” was defined later by Wolfbeis as “the system detection of physical-chemistry parameters for one use” [7].

The FOCS systems generally have three basic components:

(1) An intrinsic active sensing part (a sensor under Wolfbeis definition [2])—an active fiber (a special optic fiber, fiber or fiber optic element) with molecular/ionic recognition carried out by a transducer containing immobilized reagents, a cavity with a semipermeable membrane or micro-resonator producing an optical signal conveyed to the detection system [7]. Fibers with an IGI refraction index profile increasing the evanescence field were developed and employed in the FOCS to detect toluene in water. An OF with a xerogel layer coating of part of the PCS core in the length of some centimeters to detect aromatic hydrocarbons has been presented in [14,19,20,26,27,28,43,45,62,63],

(2) A detection system of optical signal parameters (intensity, frequency, phase) which carries the information about the monitoring parameters by sensor (refractive index, pH, concentrations of chemicals and bio-chemicals and other physical or chemical parameters) and transforms it into a useful electrical signal (current, voltage or frequency). Within the last forty years, sensitive detection systems using the principle of interference, synchronous detection of weak electric signal, which is able to detect signals under signal/noise level, have been suggested and developed. The new possibility of detection without the need of a reference beam is presented by the authors in [58]. They described the theory of detection based on an electronically phased coherence beam combination. A new sensitive silicon photomultiplier was developed with a wider linear response for the detection of weak bio-luminescence in comparison to the conventional photomultiplier tube. Distributed sensors preferably use Optical Time Domaine Reflectometry (OTDR) detection. The principle of OTDR is described by Marcuse in [5,64,65,66,67].

(3) A control and evaluation system—computer control and software for data acquisition and evaluation providing convenient service for users while measuring data in real time and on-line.

**Figure 4:**
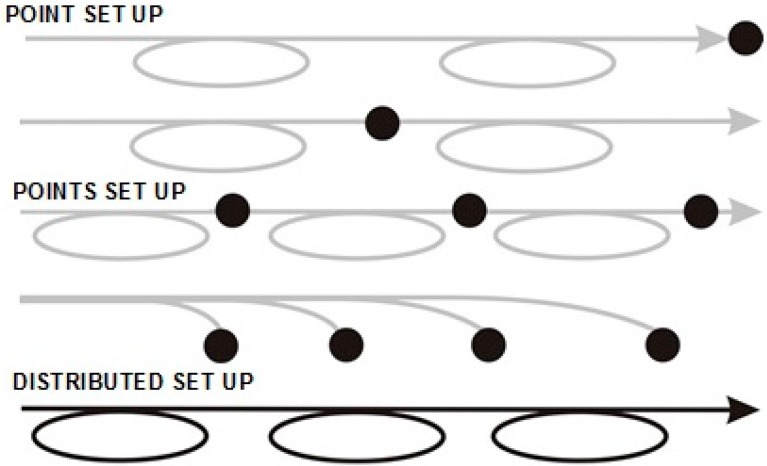
Basic scheme of FOS—Black points refer the sensing point (play) [70]. Reproduced from [47] with the permission of the CTU editors.

**Figure 5:**
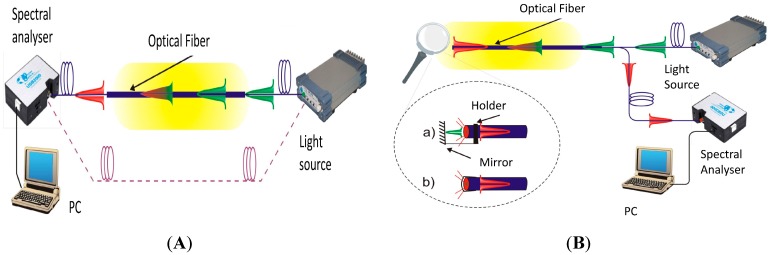
(A) Scheme of FOS in Reflection arrangement; (**B**) Scheme of FOS in Transmission arrangement. Reproduced from [47] with the permission of the CTU Editors.

The principal scheme of FOS is shown in Figure 4, the black marks refer to the active area of the sensor system. The detailed principal schemes are shown in reflection (A) and transmission (B) arrangements respectively which are shown in Figure 5. These schemes can be supplied by the reference measurement arm or the interference production system—an interferometer (Mach-Zehnder, Fabre-Perrot or Michelson configuration [5,68,69]). The quasi-distributed optical fiber sensor of pH is described in [67]. The authors described a sensor system using a 200 µm core PCS fiber with a striped part of the silicon cladding and a new coating with a polymer or sol-gel containing a transducer (indicator dye) or a polished all-silica fiber. The distributed system of the FOBS is described in Section 4.

The properties of each of the aforementioned components are responsible for the resulting parameters of the fiber optic chemical sensors (FOCS) or fiber optic biosensors (FOBS). The fundamental parameters are summarized in the following points:

(1) Responsibility—characterized by the calibration curve which shows how to change the optical signal to detected value in time. The linear part of calibration curve defines the scope of system. Obviously, it is required to be short (in seconds or minutes. Bio-sensing systems have responsibility in hours but compared to other methods this value is accepted).

(2) Sensitivity—defined as the ratio of the input to the output: $\text{σ}=\frac{dI}{dx}$ where *dI* represents a change of object (electrical signal, frequency, angle *etc*.) on a changed subject (pH, concentration of chemicals or biological material, refraction index or other physical or bio-chemical values). Obviously, it is required to be high.

(3) Reproducibility—given by the middle quadratic difference of the measured value of the chemical parameters; in other words, it is the measurement accuracy of the system.

(4) Selectivity of the system to the measured value expresses the ability of the system to significant responsibility in one or several monitoring values and a negligible response to the other.

(5) Limit of Detection (LOD)—given by the minimum value of the measured chemical parameter which can be reliably determined.

The lasers, laser diodes or diodes are usually used as a light source with a defined output intensity of the light beam, wavelength λ, spectral line width Δλ, beam diameter Φ*_B_*, beam intensity distribution *I(x*,*y*,*z)* and beam divergence φ. The laser light source is defined by further coefficients—the coherent length *l_c_* and polarization (s or p) of laser light [69]. The condition 0.7a/0.7 NA for the value of the diameter and the numerical aperture of light source beam has to be satisfied for the optimal loading of the light beam into the optical fiber defined as its radius *a* of the core and numeric aperture *NA* [71,72].

Dozens of FOCS systems have been developed combining the basic principles described in Chapter 2 and the detection systems are for synchronous detection or the interference principle of light (SPR, FBG, FLPG or interferometer arrangement). Our attention will be focused on the FOCS using the fluorescence of suitable transducers in the reflection arrangement for pH, O_2_ and CO_2_ measurement.

### 3.2. FOCS for Medical and Biological Applications

Most of those FOCS systems were developed for sensing blood gases, medical or chemical analysis or molecular biology. The most often detectable chemical parameters in this field of interest are pH, the concentration of elements (CO_2_, N_2_, K, Ca, Fe) and blood gas pressure. The FOCS use the standard spectroscopic method or less-common methods, in particular evanescence spectroscopy and specially resolved lifetime spectroscopy. Standard spectral measurement of attenuation provides information about the composition and concentration of the monitored samples. In this case, specially shaped fibers—tapered, microstructure fibers, U-shaped fibers or fiber with a sensing cladding of the OF in the transmission arrangement are preferred. The principle function and chemical composition of dye transducers (2',7'-bis-(2-carboxyethyl)-5-(and-6)-carboxyfluorescein (BCECF), 8-hydroxypyrene-1,3,6-trisulfonic acid trisodium salt (HPTS, Sigma-Aldrich, St. Louis, Missouri) *etc*.) are described in [15]. An overview of the commercially produced transducers (indicators) which are applied in FOCS can be found in [73]. The more frequently used fluorescence principle is based on the measurement of intensity dependence, intensity time dependence—life time measurement and ratio-metric fluorescence measurement [1,2,3,74]. The selection of a suitable transducer depends on the requirements of the measurement value, sensitivity and furthermore the costs of the suggested systems.

The parameters of sensor systems employing fluorescence, bioluminescence or chemo-luminescence can be improved in some of the following ways or their combination:

The development and investigation of a new type of transducers (chemical or biological) with high quantum efficiency on an inorganic base, to separate new enzymes or other recognition elements on a biological base, more stable and selective to sensing chemical or biological properties as well as new types of matrices and technologies of their immobilization or encapsulation and the application of nanotechnology.

The design of a new shape of optical fiber or optical fiber element which would increase the interrogation of light with chemicals or the application of indicators in indirect, direct measurement to increase the interrogation characteristics of the optical response light which contributes to better sensitivity. A new SPR technology with the development of thinner metal layers as one of the most suitable principles of the sensor technique.

A new detection system which would increase the signal/noise ratio by a new type of detector or new detection principle and data evaluation. A new sensitive detection method uses the interference of light. The interferometers—a comparable method using the interference of two waves from one light source, one path through a sample and a second one through the reference sample. Light speed in a sample is the function of attenuation via the refraction index of the sample (see Equation (3)). The measurement of the concentration of the sample using a configuration of the Mach-Zehnder interferometer is described in [5].

Some of mentioned ways have been described and discussed in reference papers and [75]. An important parameter in biology and medicine is pH. The FOCS developed for pH measurement often combine fluorescence principles together with an increase in the evanescent field by a tapering of the fibers. The intrinsic limitation of dye fluorescence and their dependence on ionic strength has to be taken into account. Optical nano-sensors for intracellular sensing of physiological and biological processes were provided in the publications [76,77,78]. A technique of near-field scanning microscopy using a fiber taper tip with a diameter of hundreds nm has been used to achieve fine spatial distribution. The publication [78] describes a nanoprobe using a tapered optical fiber diameter of the tip 50 nm. The tapered core was coated by metal evaporation. The whole system achieved extreme sensitivity due to the excitation in the aperture size 200 nm.

We show here an optical fiber with a sensing layer to CO_2_ and laboratory fluorescence based systems of pH sensors in the reflection arrangement employing a new design of the optical fiber element. A part of this chapter is devoted to the mathematical modeling of the shape of the optical fiber element which can amplify a weak luminescence signal. That is the case of FOCS in the reflection arrangement using a sensitive layer on the tip of element as a light source.

The material of active cladding of the OF enabling a change of its optical parameters (refractive index, color) is used in the absorption and evanescence types of FOCS. The character of the xerogel layers was measured by a contact angle in [79]. A layer from UV-curable material, consisting of organically modified polysiloxanes (ORMOCER^®^s), was coated on silica optical fibers [80]. The thin porous Si-O-Ti layer was applied on the silica core of the PCS fiber. This cladding was sensitive to CO_2_ due to the change of the absorption coefficient in the IR spectra [62,63].

The FOE (V-taper) was developed for indirect pH measurement in the reflection arrangement [81]. The drawing and photo of a tip V-taper was shown in Figure 2D. This element is fabricated by technology of the OF couplers followed by an elongation of the spliced fibers to a diameter of 5–40 µm at the temperature of a flame or focused beam of a CO_2_ laser. The element is now produced commercially by SQS Company (Nová Paka , Czech Republic) from PCS fiber or communication fibers by a standard technology of optical fiber couplers [82,83].

V-taper advantages are, as compared to Y-type couplers, summarized in the following points:

1. Homogenous excitation of the active layer immobilized on the tip element.

2. Minimized excitation intensity in the measured branch of element due to the absorption of excitation light in the active layer placed on the tip of the V-taper.

3. A possibility to increase the integration time of the detection which enables an amplification of the detection signal of fluorescence of a suitable transducer.

**Figure 6:**
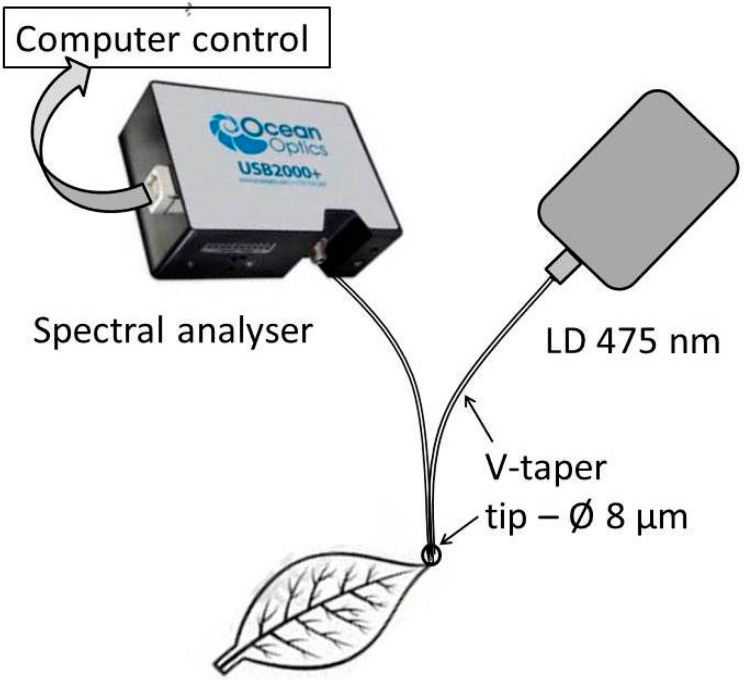
Schema of the laboratory setup of pH measurement by employing a V-taper.

**Figure 7:**
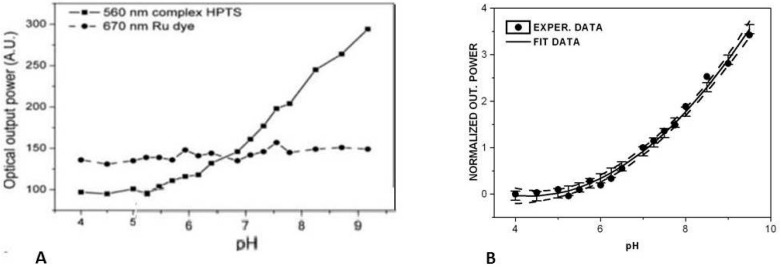
Calibration curves of the V-taper sensing probe in response to pH changes: (**A**) optical responses of the HPTS transducer and dichloro-tris-(1,10-phenanthroline)ruthenium (II) hydrate to pH; (**B**) calibration curve (including the upper and lower confidence limit dash lines [47,75]. Reproduced from [47] with the permission of CTU editors.

The laboratory setup of the pH FOCS system in plant excrement is shown in Figure 6. An HPTS transducer with a ruthenium complex, to eliminate the influence of matrices material and temperature on pH, were immobilized in silica matrix and placed on the tip of an 8 µm-diameter V-taper. The confocal microscope ZEISS LMS5 Duo (Carl Zeiss Microscopy GmbH, Jena, Germany), for a measurement of the thickness of the active layer on the tip of the V-taper based on the fluorescence of the transducer measurement as the dependence of the *z*-axis, was used [84]. The ratio-metric principle of pH measurement was applied [85]. The results of fluorescence measurement of HTPS at wavelength of 560 nm and a ruthenium complex (see Section 3) at 670 nm are shown in Figure 7 along with the experimentally determined calibration curve. The fluorescence spectra were measured by a spectral analyzer (Ocean Optics USB2000, Ocean Optics, Inc., Dunedin, FL, USA). Britton-Robinson colorless buffer solutions with different pH values but the same ionic strength were used. The buffer pH values were determined by a conventional pH meter (Jenway 3305, Bibby Scientific Ltd., Staffordshire, UK). The response time of this laboratory system was 10 s. In contrast to measurement with pH electrode, pH was measured faster and in smaller volume of plant excrement.

A V-taper with a 40-μm diameter tip and an active layer containing a ruthenium complex placed on the tip of the V-taper was tested as a possible indicator of O_2_ concentration. In this case, the light source used was a laser emitting light at 405 nm. The ruthenium complex was immobilized in a UV cured acrylate. The fluorescence at 620 nm was measured by an Ocean Optics USB2000 (Ocean Optics, Inc., Dunedin, FL, USA) spectral analyzer as a function of the qualitative changes of O_2_. The reproducibility was better than 5%.

The V taper element can be employed in fluorescence-based FOCS in the reflection arrangement with a different type of transducers immobilized in suitable matrices and placed on the tip of the element.

Another type of fiber optic element (FOE) was proposed to amplify the weak luminescence signal produced by a suitable transducer, which can be considered as a light point source, placed on its tip was shown in Figure 2C. A comparison was made with the same consideration but when OF is employed. That light source cannot be considered as a Lambertian type of light source [86,87]. The designs of the FOE shape have come out of the premise that sphere-like shaped point light sources each produce the same intensity *I_B_*_0_ of emitted luminescence into a space angle 4π. A number *N_c_* of the light source on the surface *S_OFE_* = π*d_max_* ^2^/4 immobilized in an active layer placed on the tip of the FOE is simple given by relation Equation (19)

(19) $$N_{c}=\frac{S_{OFE}}{S_{c}}$$

where *S_c_* = π*D_c_^2^*/*4* is a cross section of the light point source with a diameter *D_c_*. The mathematical model is built on searching for the optimal set of parameters *r*_0_, *A*_1_, *t*_1_, *A*_2_ and *t*_2_ and the total length *z*_max_ describing the FOE shape via Equation (20). The analytical form of the equation of the FOE shape was determined experimentally. The parameter values will be chosen from a condition to maximize the flow of light through the FOE from its broad origin that is covered by luminescence point sources to its narrower tail end with a photomultiplier detector.

(20) $$\frac{d\left(z\right)}{2}=r\left(z\right)=r_{0}+A_{1}e^{-z/t_{1}}+A_{2}e^{-z/t_{2}}$$

**Figure 8:**
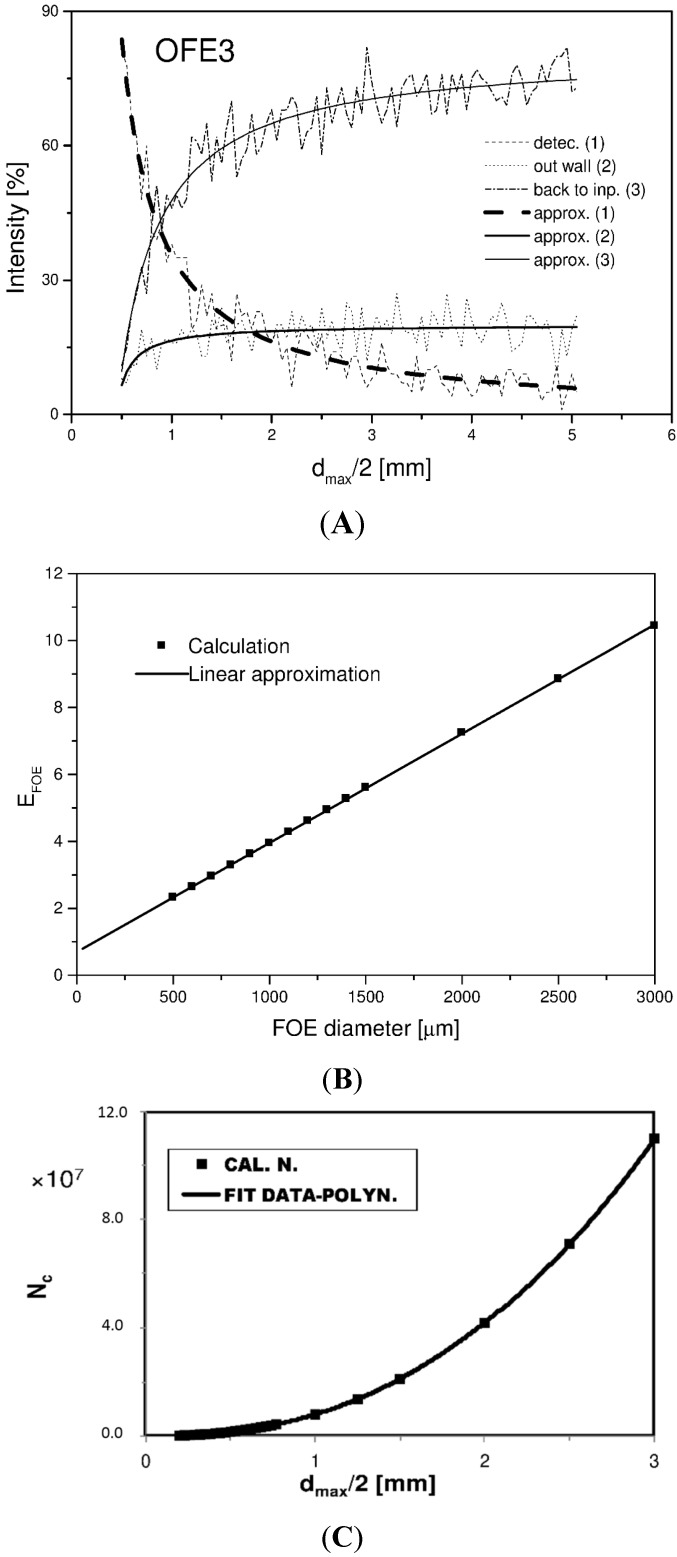
(**A**) Calculation of normalized intensity in % of ray traces by FOE 3- to detector, 2- out of wall and 1- back to impute; (**B**) Dependence of efficiency *E_FOE_* = *T_cal_(d_max_), N_c_(d_max_)*; *T* is calculated Transmission of FOE as a function of max. FOE radius; (**C**) Dependence of calculated numbers *N_C_* as a function of max. FOE radius [80].

The geometric model of the FOE is similar to a truncated cone with a non-straight line surface expressed by Equation (20). The method of ray tracing was used to find the optimal values of the FOE shape. The results of the calculations of transmission and losses are shown in Figure 8A. In this case, the shape parameters *r*_0_ = 0.42 mm; *A*_1_ = 2.12 mm; *t*_1_ = 50.77 mm and *A*_2_ = 0; *t*_2_ = 0 [85] were used. The dependence of the number of light point sources *N_c_* as a function of FOE radius for the case when *D_c_* ~ 1 μm is shown in Figure 8B.

**Figure 9:**
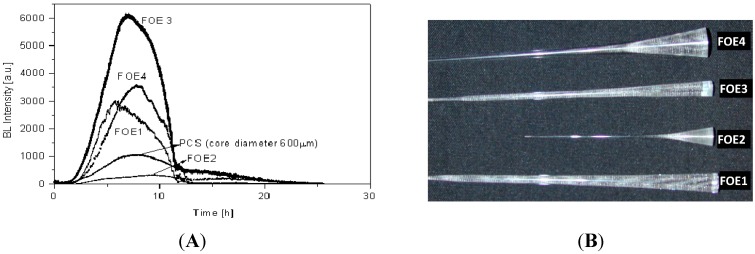
(**A**) Dependence of detected BL by FOE 1–4; (**B**) Photos of real samples of FOE produced at IPE Prague.

The results obtained from the mathematical model are in an accord with the experimental results (see Section 4.2.5). A calculated efficiency of the FOE is determined as a product of the calculated transmission and the number of light point sources for the radius of the FOE. The value of the detected BL was 5.7 × higher than with a standard PCS fiber (compare Figure 8C and Figure 9A). The influence of geometry and the shape of the FOE samples on the value of the detected luminescence are shown in Figure 9A and the photo of used samples of FOE is shown in Figure 9B. The type of FOE can be used for another application in FOS which employs a different type of light point sources immobilized in suitable matrices as the recognition elements.

## 4. Fiber Optic Biosensors (FOBS)

Based on the type of interactions with an analyte, FOBS can be divided into two groups: affinity and catalytic [8].

According to the biological recognition element used for sensing, they have four classifications:

Enzyme optical biosensors employ enzymes, which catalyze reactions with a high degree of specificity; the products or consumptions of substrates are detected either directly or via interaction with an indicator.

Whole cell optical biosensors examine the effects of an analyte on an intact cell (predominantly microbial). Optical detection is performed using an indicator (pH or oxygen) or an optical characteristic of the cells themselves as the production or vanishing fluorescence or bioluminescence.

Immunoassay optical biosensors make use of the specific binding between an antibody and antigen. Binding is monitored via a fluorescent label or by observation of a refractive index or reflectivity change, which requires no label.

Nucleic acid optical biosensors employ the affinity of single-stranded DNA (ssDNA) to form a double-stranded DNA (dsDNA) with complementary sequences. These sensors typically require labeling of one member of the ssDNA with an optical indicator.

**Figure 10:**
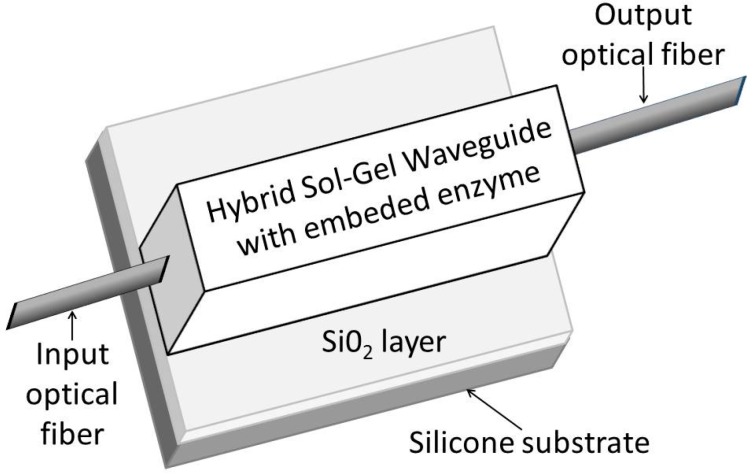
Bio-doped polymeric strip waveguide (Adapted from [94] with permission from the European Society for Photobiology, the European Photochemistry Association, and The Royal Society of Chemistry).

According to their mode of action, optical biosensors have been subdivided into five subgroups [88]: (a) plain fluorometric sensors; (b) direct and indirect indicator-mediated chemical sensors; (c) direct enzymatic biosensors; (d) indicator-mediated enzymatic biosensors; and (e) affinity biosensors. The optical measurement method of fiber biosensors is the same as described (for fiber chemical sensors) in chapter 3. The typical methods include absorbance or reflectance, fluorescence, bioluminescence or chemiluminescence. Optical fiber based catalytic biosensors are often extrinsic sensors. In these sensors, an optical fiber transmits light from a biorecognition element immobilized on the polished end of the fiber. Intrinsic sensors, mostly affinity sensors, have a biorecognition element bonded on the surface of the fiber core and their electro-optical instrumentation interrogate evanescent or plasmon waves [89,90]. The principle of fluorescence resonance energy transfer (FRET) utilized fiber optic biosensors for the fast detection of *Salmonella typhimurium* [91]. Monitoring of protein adsorption with a Fabry-Perot interferometric fiber-optic method [88], SPR [92] or PCF interferometry [93] is more advantageous as compared with other methods. Bio-doped polymeric strips of a waveguide configuration (Figure 10) were fabricated by micromolding in capillaries. In contrast to evanescent field sensors, the sensor response does not only rely on the interaction of the evanescent wave with the recognition element, but on the interaction of the whole field. The potential of this approach was demonstrated by the development of a biosensor for the detection of H_2_O_2_ using the enzyme horseradish peroxidase as the doping agent [94]. Long-term plastic optical fiber sensors have been described by Wong *et al.* [95]. The sensor monitored biofouling via the evanescent field. Biofilm is a dense community of microorganisms (predominantly bacteria) stick together by a extracellular polymers. It consists of 98% water, and is transparent, with a refractive index (RI) of 1.336. This is only 0.15% higher than water (1.334) at RI. The sensitivity of the evanescent field biofouling sensor was ±0.007 RI. The application of POF evanescent field sensor was proposed as an earlier warning of biofouling in water systems and heat exchangers [96]. Tapered plastic optical fiber-based biosensor for cells detection were constructed in U-shaped (bended) configurations, with taper waist diameters ranging from 0.40 mm up to 0.50 mm [23]. Low cost surface plasmon resonance (SPR) biosensor, based on the utilization of plastic optical fibers was used to monitor the formation of the transglutaminase/anti-transglutaminase antibodies for diagnosis of celiac disease [97].

Single-core fiber-based sensors have been used to detect individual analytes in complex samples. Optical fiber arrays (OFA) enable simultaneous, multi-analyte, high-density detection. OFA have been prepared using two different approaches [98]. In the first approach, multiple single-core fibers, each modified with a different sensing chemistry using the techniques employed for preparing single-core fiber sensors, are bundled to create multi-analyte sensors. The second approach is based on optical fiber bundles that comprise thousands of individual single-core fibers, each surrounded by cladding material, coherently fused together. Each fiber in the fused bundle can be modified with a different sensing chemistry using a random assembly method. In this approach, the chemical etching of the optical fiber cores is used to form micron-sized wells at the distal end of the fiber, with the dimensions of the wells defined by the cladding surrounding the individual cores. Beads containing biological recognition elements (DNA and protein) are then loaded into the microwells. Living cells have also been loaded in the wells for multiplexed sensing [99]. DNA microarrays employing microsphere-immobilized oligonucleotides were used to develop a cross-reactive multi-locus sequence typing-based method for the characterization of closely related *E. coli* strains [100]. Various assay formats of the fiber optic immunosensors and nucleic base fiber optic sensors are the basis for multiplexed pathogen detection systems [101]. A tapered optical fiber bundle was used to contact a disposable microwell cartridge (scheme see also Section 4.2.6) with immobilized whole-cell biosensors with a compact charge couple device (CCD) sensor [102]. A pH sensors array (7 dots Ø 92 µm) of photopolymerizable sensing elements on optical fiber (Ø 500 µm) was fabricated with a microjet printing process. This fabrication technique prevails over dip coating in terms of reproducibility with minimum cross sensitivity [103].

### 4.1. Enzymatic Optical Fiber Sensors with an Oxygen Transducer

The abundance of optical fiber enzymatic biosensors reported in the literature (reviews [3,104,105,106]) is likely due to the inherent selectivity and the fact that they resemble chemical sensors, as an enzyme might be viewed as a “catalyst with a big molecule”. Among optical fiber enzymatic sensors, by far the greatest attention has been paid to sensors of glucose as they are promising tools for the on-line *in situ* control of the glucose level *in vitro* and possibly *in vivo* [107].

Currently, most glucose biosensors utilize glucose oxidase as their recognition element that catalyzes the oxidation of glucose to gluconolactone:

Glucose + O_2_ → gluconolactone + H_2_O_2_ (21)

Glucose oxidase is one of the enzyme oxidases which react with molecular oxygen to catalyze the oxidation of a substrate. Oxygen consumption could be easily monitored by an oxygen electrode or optode.

Since 1974 when Hesse [108] described the first optical fiber fluorescent oxygen sensor, a wide variety of designs, applications and chemical sensors using an optical oxygen sensor as the transducer have been performed [109,110,111]. At present, the optical sensors of oxygen and likewise their optical fiber variations are commercially available (e.g., Ocean Optics) and autoclavable [112]. The measurement is based on the fluorescence quenching of metal complexes. Colorimetric-based measurement is also possible [113,114]. In contrast with their electrical alternative—Clark electrode—oxygen optodes do not consume oxygen and their signal is independent of mixing. Thanks to these features, optical sensors are particularly suited for measurement in biological fluids that are often viscous and oxygen consumption is undesirable during the measurement.

The design of an optical fiber oxygen sensor oftentimes comprises oxygen sensitive chemistry placed in front of the OF through which exciting light is guided. The fluorescence emitted is guided back through the same or another OF or a bundle. Measurement of the dynamic quenching affected by oxygen is based either on the fluorescence intensity or decay time.

Life is often accompanied by oxygen utilization. Oxidases are enzymes reducing the activation energy of oxidation-reduction reactions and involving reduction of oxygen to water or hydrogen peroxide. Thus at constant content of an enzyme oxidase the consumption of oxygen is proportional to the concentration of the substrate. In real sensors, oxidases are immobilized on the surface of the oxygen transducer such as a membrane of a Clark electrode or in the case of an optical sensor on a membrane with an immobilized fluorescent metal complex. Without transducers, optical sensors with oxidases based on the interrogation of the intrinsic fluorescence of enzyme needed instrumentation with a femtosecond resolution [115].

Oxidases have been employed in the analytical methods of the determination of biologically important compounds for 60 years. The list of sensors with oxidases includes sensors of alcohols, ammonia, glucose, glutamate, cholesterol, lactic acid, lactose, uric acid, urea, purine derivates, catechol amines, biogenic amines, gamma-amino butyric acid [116], phosphate, nitrate, sulphate [117], hydrogen peroxide, phenol [118], superoxide and nitric oxide radicals. Many of these sensors have been demonstrated with amperometric or potentiometric transducers. Fluorescent sensors of both oxygen and pH [119] have served as optical transducers. The fiber optic H_2_O_2_ sensor was combined with packed bed enzyme reactors to determine, e.g., glucose, lactate, glutamine, glutamate, ammonia, xanthine, hypoxanthine and phosphate in the range between 10^−3^ and 10^−7^ mol/L [120]. Fiber optic biosensors based on fluorescence measurements were reviewed by Bosch in 2007 [4]. Recently, enzymatic sensors of toluene and adrenaline with optical oxygen transducers have been reported. The sensor of toluene is based on the oxidation of toluene catalyzed by mono-oxygenase. The sensor of adrenaline [121] employed an enzyme laccase immobilized on the copper tetra-aminophthalocyanine magnetite composite nanoparticles and the fluorescent oxygen-sensing membrane. Yucca filamentosa plant leaf membrane was used instead of a separated enzyme catalase in the construction of a hydrogen peroxide biosensor with excellent stability, in 2364 measurements [122].

Despite the principles of FOBS had been demonstrated long ago, their use outside of laboratories has remained rare. From the point of view of robustness and operational stability at the current stage of development, the reliability of optical fibers and electro-optical components is high but the weakest link is the biological component and its binding to a transducer.

Many immobilization techniques and polymers have been used to immobilize oxidases on optical and electrical transducers. Among them, the easiest procedure is crosslinking with glutaraldehyde and entrapment in organic-inorganic matrices that impart a higher chemical and mechanical durability to the recognition elements. Effective glutaraldehyde crosslinking requires a rinse step and a 24-h incubation step to minimize enzyme losses [123]. An enzyme entrapped in a silica gel via a sol-gel process could sustain high activity and its thermostability might be improved. However, an enzyme leaked from a loose matrix and its opposite in matrices with small pores that prevent enzyme leakage, enzyme activity is reduced due to the limited transport of substrates and products in the small pores. A reliable and fast method of the determination of pore dimensions in a watery silica gel is needed for a suitable design of a sol-gel silica matrix. It is believed that a rational combination of various immobilization methods is a valuable approach to obtain a robust immobilized enzyme that cannot be obtained by straightforward immobilization [124].

In the design of a fiber glucose subcutaneous sensor, the leakage of glucose oxidase, cross-linked with glutaraldehyde, is prevented by the membrane, composed from cellulose and polyurethane [125]. Protection against enzyme leakage by a membrane or entrapment in a matrix with small pores basically determines the linear range of the sensor [126]. The dynamic range up to 20 mM of glucose was obtained by varying the thickness of the outer membrane of the subcutaneous glucose sensor [125].

**Figure 11:**
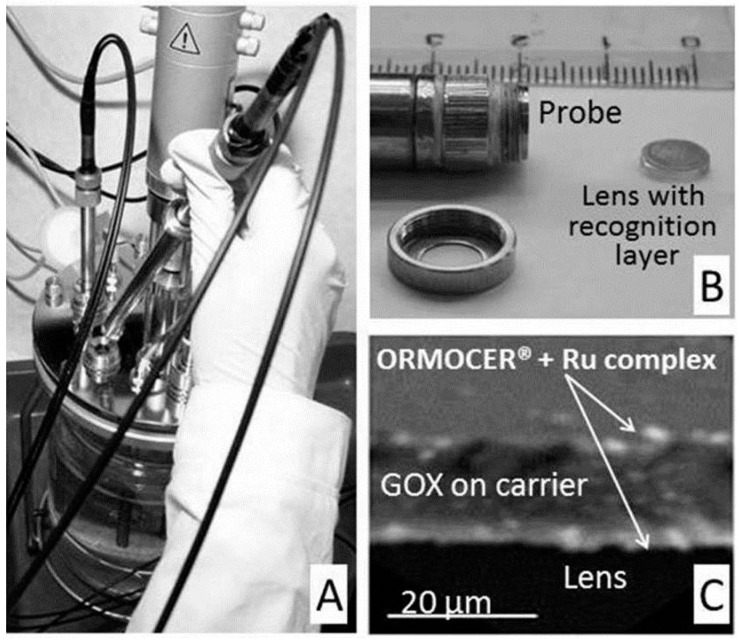
A bioreactor equipped with oxygen and glucose optical fiber probe (**A**) optical fiber probe; (**B**) SEM of the recognition layer composed from ORMOCER^®^ with a ruthenium complex (**C**).

**Figure 12:**
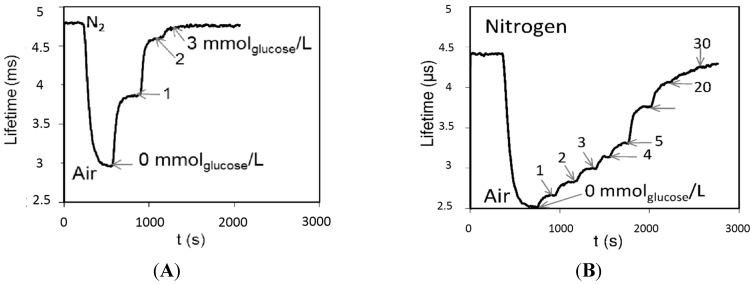
Optical fiber sensor of glucose for bioreactors. Record of lifetime measurement (**A**) sensitivity of the probe 20 μmol/L and linear dynamic range 2 mmol/L; (**B**) sensitivity of the probe 200 μmol/L and dynamic range 30 mmol/L.

**Figure 13:**
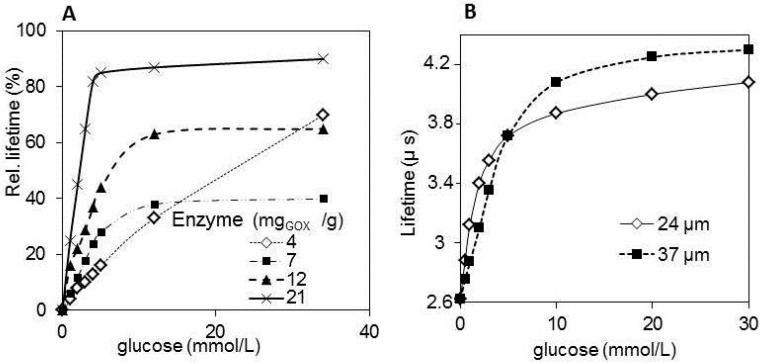
Optical fiber sensor of glucose for bioreactors. The influence of thickness of recognition layer on the sensor response (**A**); The influence of Glucose oxidase enzyme (GOX, E.C. 1.1.3.4.) content (Enzyme loading on the carrier: 75 mg GOX/g Sepabeds^®^) (**B**).

In the case of a glucose sensor for bioreactors (Figure 11), the durability and mechanical stability against abrasion in a mixed bioreactor was ensured by the interconnecting polymer ORMOCER^®^ [127]. In this sensor, covalent attachment to the macroporous synthetic polymer carrier protects the enzyme against harsh conditions after mixing with ORMOCER^®^ and during the UV curing of the sensitive layer on the acrylate lens. The sensitivity and dynamic range was influenced by the thickness of the layer (Figure 12 and Figure 13), the enzyme content and porous forming components in broad ranges of sensitivity from 2 to 200 µmol/L and a dynamic range from 2 to 30 mmol/L [128,129]. The SEM (Scanning electron microscopy) image of the layer (Figure 11C) revealed that the sensitive layer looks like a sandwich, with a porous carrier and an attached glucose oxidase being caught between two ORMOCER^®^ sheets. The sensor was sterilizable with ethanol, isopropanol or UV light [130]. Over one week of bioreactor fermentation, the response of the sensor was stable and was not influenced by dissolved oxygen fluctuation provided that the air saturation did not drop below 80%. In fermentation media and beverages, the response of the sensor was influenced by oxygen solubility. The utilization of magnetic carriers made the preparation of the sensor layer easier as was demonstrated with the sensor of biogenic amines [131]. Enzymatic optical fiber sensors with an oxygen transducer were demonstrated as a viable scheme of chemical sensors for medical [125] and industrial application [132]. The dynamic range of the glucose sensor for bioreactors reached at most one third of the full range for industrial application, which is 0–15 g/L [129]. A further disadvantage is calibration dependent on the composition, which might be compensated by the simultaneous measurement of dissolved oxygen. Nevertheless, the sensitivity, durability and the dynamic range of developed sensors meet the demands for the determination of remnants of glucose in diet beverages and the control of lower glucose content in bioreactors. UV-cured polymers were applied also in preparation of microscale fluorescence lifetime based optrodes, which showed linear response within the physiologically relevant range of oxygen concentrations as well as fast response times [133].

### 4.2. Whole Cell Optical Fiber Sensors

**Figure 14:**
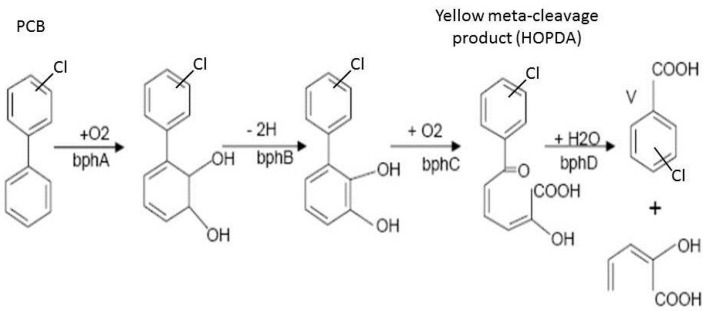
Polychlorinated biphenyls (PCB) degradation pathway by *Pseudomonas sp*. P2 (Adopted from [137]).

In contrast to enzymatic sensors, which are endowed with high selectivity, and therefore their responses correspond to chemical sensors, whole cell sensors exhibit rather broad selectivity [134,135,136]. Nevertheless, whole cell sensors are advantageous if the detection needs a sequence of multiple enzymatic reactions, which are difficult to accomplish *in vitro*. An *in vivo* cell enzymatic system easily transforms the analyte into an optically detectable product. A typical example is a whole cell sensor of polychlorinated biphenyls (PCB) based on the detection of yellow products as a result of PCB biodegradation in a sequence of enzymatic reactions [137]. Aerobic bacteria, isolated from contaminated soil, co-metabolically transform PCBs to chlorobenzoic acids through four enzymatic reactions (Figure 14). Two reactions are catalyzed by dioxygenases (bphA, bphC). The second step is catalyzed by dehydrogenase (bphB) and the last reaction is catalyzed by hydrolase (bphD). The third step of this biphenyl catabolic pathway generates the corresponding chlorinated 2-hydroxy-6-oxo-6-phenylhexa-2,4-dienoic acid (HOPDA), a yellow meta ring-fission metabolite that absorbs light with its absorption maximum at λ = 398 nm.

A sensor based on measuring of the cellular response to exposure to various factors provides data that differs in quality from the data collected by analytical sensors that measure the physical and chemical factors directly: the magnitude of the effect of a factor on life is measured as opposed to the quantity or presence of the factor itself. In contrast to chemical analysis by chromatographic methods that measure only the total concentration of a target analyte in a sample, whole-cell biosensors possess the ability to indicate bioavailability, which indicate that the analyte can be assimilated by or directly effects a living organism [138,139,140,141,142]. By employing living cells as sensors, bioanalytes can be screened without requiring a priori knowledge of the analyte’s chemistry. Whole-cell biosensors detect a variety of biological, organic, and inorganic compounds and can produce a detectable outputs that can be easily quantifiable, for example, fluorescent proteins measured by a fluorometer, dyes and pigments measured by spectroscopy and by the human eye, and electrochemical signals such as pH measured by litmus paper or a pH meter. These easily measured outputs require little expertise to use or to interpret. Microbial sensors have used amperometric, chronoamperometric, voltammetric, conductometric, impedimetric and optical transducers [143]. Although many cell-based assays have been described, for environmental monitoring, food quality assurance and antidoping screening, few can be regarded as true biosensors [144]. OF are considered to be ideal transducers for whole cell sensors [145]. Microorganisms applied as the recognition elements of FOS include both wild and genetically engineered microorganisms.

#### 4.2.1. Biosensors of Biochemical Oxygen Demand (BOD)

Wild microorganisms, including yeast [146], bacteria and mixed cultures [147,148] have been employed in the design of instruments for the measurement of Biochemical Oxygen Demand (BOD), which is a gauge of the effectiveness of wastewater treatment plants. BOD is the amount of oxygen required for microbial metabolism of organic compounds in water. The traditional bioassay method for estimating the BOD_5_ involves the incubation of sample water for 5 days. BOD biosensors, which shorten the time needed for analysis to minutes, are based on an amperometric oxygen sensor and biomembrane that consists of immobilized mixed culture of microorganisms [149]. They employ electrical oxygen transducers (Clark electrodes) but fiber optic oxygen transducers (e.g., Ocean Optics) are also available. BOD biosensors have several limitations such as the need for reactivation, and the inability to respond to changing quality characteristics of wastewater. The reproducibility of the result and the uncertainty associated with the calibration function for translating the BOD substitute into the BOD_5_ are influenced by diffusion processes of the biodegradable organic matter into the membrane and different responses by different microbial species [150,151]. An overview of the preparation of biorecognition elements, immobilization techniques and various applications of optical fiber BOD sensors used as components of devices for monitoring wastewater pollution have been reported [152,153,154]. Optical fiber BOD sensors have a response time in minutes and BOD values correlated with those determined by conventional BOD_5_ methods [155]. However eventual implementation in practical devices needs long term maintenance of both the viability and activity of microorganisms. To meet these demands, various techniques of microorganisms preservation and fixation have been reported including freeze drying, vacuum drying, continuous cultivation and entrapment in organic and inorganic polymers [148]. But none have surpassed the obstacles for utilization in a practical device [156,157].

#### 4.2.2. Biosensors of Pollutants and Toxic Compounds with Natural Cells

Free bioluminescent bacteriium *Vibrio fisheri* has been base of the most widespread acute toxicity test (e.g., MICROTOX). Biosensors with *Vibrio fisheri* have not been exploited, because immobilization significantly influences the highly sensitive production of the bioluminescence of this bacterium. The cyanobacteria *Anabaena torulosa* entrapped on a cellulose membrane and fixed into a cylindrical well connected to a fluorescence spectrometer with OF indicated the presence of heavy metals (Cu, Pb, and Cd), 2,4-dichlorophenoxyacetate, and chlorpyrifos. When the organisms are exposed to toxicants, major photosynthetic transport pathways are inhibited. Thus, the fluorescence emission will increase as a way to diffuse the energy, which has been absorbed. The presence of the toxicants was indicated by the change of fluorescence emission, before and after the exposure [158].

Bacteria, isolated from sewage waste, producing asparginase, were co-immobilized with the pH indicator in a sol-gel film to construct miniaturized FOBS for monitoring l-asparagine. Its content is proportional to acrylamides, which pose health risks, as they act as a neurotoxin and carcinogen in humans. The enzyme asparginase hydrolyzes l-asparagine to form l-aspartic acid and ammonia, and the associated increase in local pH results in a change of the absorption spectrum of the pH indicator. The biosensor has an operational range from 0.1 M to 1 nM and a storage stability of 40 days. The biosensor was applied to quantify L-asparagine in beverages [159].

Membrane containing microalgae encapsulated in a matrix from sodium silicate and glycerol was placed at the tip of a bifurcated fiber-optic cable in a flow-through cell to form a biosensor of herbicides. In the presence of herbicides, chlorophyll fluorescence is increased because the herbicides destroy the photosystem. A biosensor works in concentrations from 0.5 µg/L to 10 mg/L with a partially reversible response [160]. The same authors used microalgae to construct an optical fiber device for monitoring copper in reservoirs and water supplies [161].

*Pseudomonas sp.* P2, selected from polychlorinated biphenyls (PCB) contaminated places, which degrade 3-chlorobiphenyls (3-CB) evolving a yellow stable metabolite (Figure 14) were co-entrapped with biphenyl (an essential co-substrate of PCB co-metabolism) into a silica matrix to form a semiquantitative sensor of PCB. Light absorbance (λ = 400 nm) of the medium was proportional to the PCB concentration with detection limit of 0.2 mg 2,4,4'-trichlorobiphenyl/ L. The storage stability was 2 weeks [137]. The sensitivity of this type of sensor was substantially increased by the application of a liquid core waveguide [162].

The toxic effects in microorganisms have a limited predictive value for possible hazards for humans and these biomonitors do not react to the non-systemic, specific toxic effects of compounds such as genotoxicants and endocrine disruptors. The disruption induced in living mammalian cells by toxicants were interrogated in human lung cells attached as the cladding of a chalcogenide glass fiber (Te_2_As_3_Se_5_ Ø 400 μm) A sensing zone, about 10 cm long was tapered to diameter of 100 µm. The cytotoxic detergent Triton X-100 that dissolves the membrane by destroying the phospholipid bilayer caused a rapid alteration of the spectrum (completed within 20 min), which corresponds to the methylene and methyl vibration of the membrane phospholipids. The affect of the genotoxic agent, etoposide that induced DNA cleavage and cross linking, caused changes of the spectrum in the amino-acids region [163].

A multi-well plate-based biosensor containing mammalian cells B-cell hybridoma, Ped-2E9, encapsulated in type I collagen matrix, was developed for the rapid detection of the viable cells of pathogenic *Listeria*, the toxin listeriolysin O, and the enterotoxin from the *Bacillus* species. This sensor measures the alkaline phosphatase release from infected Ped-2E9 cells colorimetrically [164]. An optical fiber version of such a sensor is conceivable. Likewise, OF could be employed in an electrooptical instrumentation of a sensor with smooth muscle cells immobilized in nanoliter volume collagen droplets by the printing technique. The cell response to external stimulus as reflected by cell morphology, (*i.e.*, cell spread size, changes of directionality of spread cells and their relative positions) was imaged using a lensless CCD. In addition to the detection of minor changes in the cell environment, the method to determine cellular directionality has important applications in tissue engineering to generate tissue constructs that are mechanically functional, since the aligned muscle bundles are shown to better mimic the native tissue structure [165].

#### 4.2.3. Multi-Wavelength Fluorescence Spectroscopy of Microorganisms

**Figure 15:**
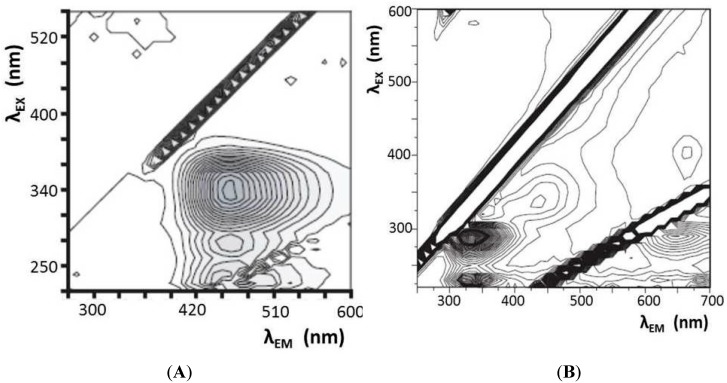
2-D fluorescence spectrum (**A**) nicotineamide dinucleotide (NADH) (pH = 7) and (**B**) *Sacharomyces cerevisiae* SP4 immobilized in alginate.

A special method of whole cell biosensing that utilize OF but do not need cell immobilization or staining is the monitoring of the indigenous fluorescence of microorganism. 2D-fluorescence spectrum is a matrix of excitation (λ_ex_) and emission (λ_em_) wavelengths, and the intensity of fluorescence. The maximal intensity of the fluorescence of individual fluorophores is determined by the coordinates λ_ex_/λ_em_. Biological processes depend on proteins, which fluoresce because of fluorescent amino acids such as tryptophan (λ_ex_/λ_em_ = 275/303 nm, pH = 7) tyrosine (287/348 nm), phenylalanine (260/282 nm). In another region of the fluorescence spectra, there are GFP (490/518 nm) vitamins e.g., pyridoxine, riboflavin and coenzymes; reduced forms of nicotineamide dinucleotide (NADH), nicotineamide dinucleotide phosphate (NADPH), flavine adenine dinucleotide (FAD) and flavine mononucleotide (FMN). The 2D-spectrum of NADH (340/460 nm) is depicted in Figure 15A. Spots of fluorescence maxima of biogenic fluorophores are visible in the 2D-spectra of microorganisms, in both suspended and immobilized cultures, such as the yeast immobilized in alginate seen in Figure 15B. Multiwavelength scanning of a culture of over the UV-VIS-NIR range of excitation and emission wavelengths is an effective tool of the monitoring of bioreactor processes. 2D-spectrofluorometer BioView^®^ connected via bifurcated optical fibers with a bioreactor have been applied for the estimation of substrate consumption as well as the formation of low molecular weight products and the cell biomass of industrially important bacteria and fungi [166,167]. The measurement of NAD(P)H fluorescence intensity and its changes during oxygen depletion was used to determine the concentration of active immobilized biomass [168,169].

#### 4.2.4. FOBS with Genetically Engineered Bioreporters

Bioreporters are genetically engineered microorganisms designed for the detection of chemical, physical or biological signals via the production of a suitable reporter protein, such as luciferase, β-galactosidase or autofluorescent proteins. Bioreporters enable the monitoring of metabolic gene expression (mostly regulated via induction or repression) by a measurement of the signal resulting from the joint expression of linked reporter genes. The majority of bioreporters utilize reporter genes for enzyme activities linked to the generation of light, for example, fluorescence (green fluorescent protein (GFP) and its differently colored variants [170]) or bioluminescence (bacterial (*lux*) or insect (*luc*) luciferases) signaling outputs [134,171].

Important application of whole-cell biosensors is pollution monitoring in the environment [172], where the bioreporters must face samples, consisting of complex mixtures of compounds. Carbon assimilation in bacteria generally follows a hierarchy, where certain carbon sources are preferentially consumed over others. The presence of additional carbon sources in natural samples and general growth conditions could therefore interfere with the correct performance of the reporter system in the field.

Clonal bacterial populations are essentially always physiologically, phenotypically and genotypically heterogeneous, thus emphasizing the need for sound statistical approaches for the interpretation of the reporter response in individual bacterial cells [173].

#### 4.2.5. FOBS with Microorganisms Expressing Green Fluorescent Protein (GFP)

GFP is a member of the family of proteins capable of light fluorescence in a variety of colors. Wild-type GFP protein has excitation maxima at two wavelengths λ_ex_ = 397 nm and λ_ex_ = 475 nm and one emission λ_em_ = 504 nm [170]. Since GFP was first expressed in *E. coli* it has been used in many biosensors using the induction or suppression of GFP expression to indicate interaction with an analyte of interest.

One of the more popular assays focusing on the suppressed expression of GFP is the determination of cell viability. Bacteria expressing GFP are exposed to compounds of interest and the severity of the toxicity is determined by monitoring the decrease in fluorescent expression. As the bacteria are killed or their metabolism is slowed by interaction with toxic substances, the overall amount of fluorescence will decrease [171].

The inverse of this type of experiment is to induce the expression of GFP as a positive result. In this case, the gene encoding for GFP is placed under the control of a genetic promoter that responds specifically to the analyte of interest. An advantage of this type of experimental design is that the amount of fluorescence produced can be correlated to the concentration of the analyte, allowing for an approximate quantification [171].

Shetty *et al.* [174] constructed a GFP-based *E. coli* sensitive to the monosaccharide l-arabinose. Bacteria were entrapped within a dialysis membrane on the tip of a fiber optic bundle connected with a tungsten lamp and photomultiplier (PMT). The immersion of the *E. coli* entrapped sensor end of the fiber bundle in liquid then allowed the detection of L-arabinose at varying concentrations. To improve sensitivity, Knight *et al.* [175] bypassed fiber optics by interfacing a PMT directly with a flow-cell containing a yeast-based GFP bioreporter sensitive to DNA damage by genotoxic compounds. The use of a fluorescence polarization approach enhanced the detection of GFP expressed by the genetically modified yeast cells in the presence of cellular autofluorescence, when compared to a conventional fluorescence method [176].

GFP is very stable thus its fluorescence does not disappear immediately after cell death. The accumulation of GFP, often viewed as a hindrance to the interpretation of expression data, was analyzed by taking the time-derivative of fluorescence intensity. Comparisons of the fluorescence intensity derivative curves with the timing of induction and administration of protein synthesis inhibitors revealed GFP’s utility in near real-time measurements of fluorescence induction [177].

#### 4.2.6. FOBS with Immobilized Living Bioluminescent Bioreporters

Bioluminescent bioreporters were originally constructed as whole-cell bacterial biosensors that respond to specific chemicals or physical agents in their environment via the production of visible light [178]. Bioluminescent bacteria express luminescence through the production of luciferase, either bacterial (*lux*) or firefly (*luc*). The latter has the advantage of a higher quantum yield, but requires the constant addition of luciferine. Bacterial luciferase catalyzes the oxidation of a long-chain aliphatic aldehyde (RCHO) and a reduced flavin mononucleotide (FMNH_2_). In this reaction, free energy is emitted in the form of light with a wavelength of 490 nm:

FMNH_2_ + RCHO + O_2_ → FMN + RCOOH + H_2_O + light (22)

As this reaction depends on a functional electron transport system, it only functions in viable cells. The *luxAB* genes, coding for lucipherase, are the minimum required for luminescence; however, in this case a substrate has to be administered externally. The use of the *luxCDABE* genes, in which *luxCDE* code for the (re)generation of the substrate, is more practical for online monitoring. Thus, no substrate addition is necessary and the luciferase reporter can operate independently. Such organisms utilized a genetic construct consisting of the lux cassette derived from the marine bacterium, *Vibrio fisheri*. The *lux* cassette consists of five genes, *luxA*, *B*, *C*, *D* and *E*. Coupling the lux cassette to an inducible promoter gene generates a bioreporter capable of generating visible light in a target-specific manner, with no requirement for the extraneous addition of substrate [179,180].

Currently the factor limiting the application of bioreporters is the fact that they are genetically modified. In developed countries as EU and USA, any activity with genetically modified organisms (GMO) is strictly regulated. Applicants apply for GMO authorizations by submitting a dossier with experimental data and a risk assessment. In EU the dominant number of genetically modified microorganisms (GMM) are authorized for laboratory use only (“contained use”) [181] under the EU Directive 2009/41/EC [182]. Anyone planning to commence contained use activity must notify its competent authorities which verify that the installation is appropriate for the activity and that the work does not pose any danger to human health and the environment. Standard methodology for use of genetically engineered bioluminescent bioreporters for detection of contaminants in real environment (water and soil samples) was described [183]. If we detach the general public fear from GMO, the lack of knowledge of environmental fate of engineered bacteria and spread of their genes is one of the strong reasons for such practice.

In 1996, the first EPA-sanctioned release of a recombinant microbe (*Pseudomonas fluorescens* HK44) into the subsurface soil environment was initiated [184,185]. With an aim to access the survivability/environmental fate of HK44, soil sampling was performed 14 years post release. Although after extensive sampling, culturable HK44 cells were not found, qPCR and metagenomic analyses indicated that the genetic signatures of HK44 cells still persisted in the soils, with genes diagnostic for the bioluminescent transposon carried by strain HK44 (*luxA* and *tetA*) being found at low concentrations [186].

The *lux* bioreporter organism *Pseudomonas putida* RB1353 was employed in a fiber optic detection system for non-invasively monitoring of real-time, *in situ* microbial activity in porous media. The system including optical fiber for the transmission of bioluminescence to photomultiplier and fluid sampling with oxygen electrode effectively captured the dynamics of *in situ* bacterial gene expression during naphthalene catabolism under changing physicochemical conditions in a saturated porous media. The system was not adversely affected by biofilm formation on the optical fiber tips (fiber with polymethyl methacrylate core Ø 2.6 mm and a fluorinated polymer cladding NA = 0.5) or by bioluminescence attenuation in the porous medium employed [187].

The unique specificity of bacteriophage (bacterial viruses) has been exploited in lux bioluminescent assays for specific identification of foodborne bacterial pathogens such as *E. coli* O157:H7. The potential exists for immobilization and the development of optical fiber sensors [188].

**Figure 16:**
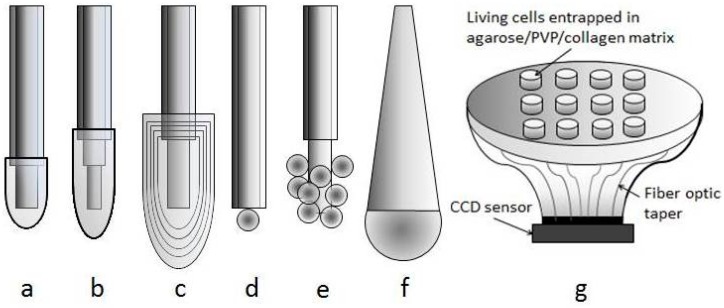
Whole cell optical fiber biosensors. Scheme of cell fixations on the tip of an optical fiber; cells entrapped in alginate (**a**–**c**) [189]; Biotin-alginate microspheres conjugated to an optical fiber via avidin-biotin affinity interactions (**d**,**e**) [189]; cells entrapped in a silica gel on the end of tapered up quartz fiber (Ø 5 mm) (**f**) [166]; microwells with cells entrapped in a polymer mixture on a fiber optic taper (Ø 27 mm/Ø 11 mm) (**g**) [102].

In laboratories, the light response generated by the bioluminescencent bacteria is tested in liquid suspension. To employ bacterial bioreporters as real field sensors the bacteria were immobilized and combined with a hand-held photomultiplier, integrated circuit luminometer or optical fibers. The first bioluminescent bioreporters tied on the tip of OF were entrapped in alginate [184,189]. The scheme of microorganisms entrapments on the tips of OF with immobilized bioluminescent bioreporters are on Figure 16. The cells were entrapped in alginate (an instrument for simultaneous coating 22 fiber ends were developed [190]), alginate—biotin microbeads, in alginate layers [189] and in silica gel on the down tapered OF [191]. The recognition element of the biosensor of mitomycin C was prepared by conjugating the biotinylated alginate microspheres to the surface of a streptavidin-coated quartz optical fiber with a diameter of 1000 µm (in Figure 16d,e). As a compromise between the need for a high percent of molar modification of the alginate, on the one hand, and sufficient gelling capability, on the other hand, an optimal modification of 10%–13% of biotin-alginate was used [192].

The optimal response of the same biosensor with an alginate layer recognition element was achieved with six alginate/bacterial adlayers on a 1 cm exposed fiber-optic core (pure silica core diameter of 400 μm, with a refractive index of 1.4571 (λ = 633nm) and a cladding diameter of 440 μm, with a refractive index of 1.4011 (λ = 633 nm) (Figure 16a,c). The total alginate volume per tip was about 100 μL, containing a bacterial suspension of around 1.5–3.0 × 10^7^ cells. When the core diameter was etched down from 400 to 270 μm (Figure 16b), the photon detection efficiency increased (by roughly twofold), although to a lesser extent than that expected from the theoretical calculations [189].

The *Pseudomonas putida* TV1601 strain entrapped in alginate responded to chloroform concentrations that were lower than US permission limits (50 ppm). This alginate entrapped bioreporter on the tip of 400 μm core of OF was incorporated in a portable FOBS and the authors predict that one day it could be used as an air pollution alert system in an indoor environment [193].

**Figure 17:**
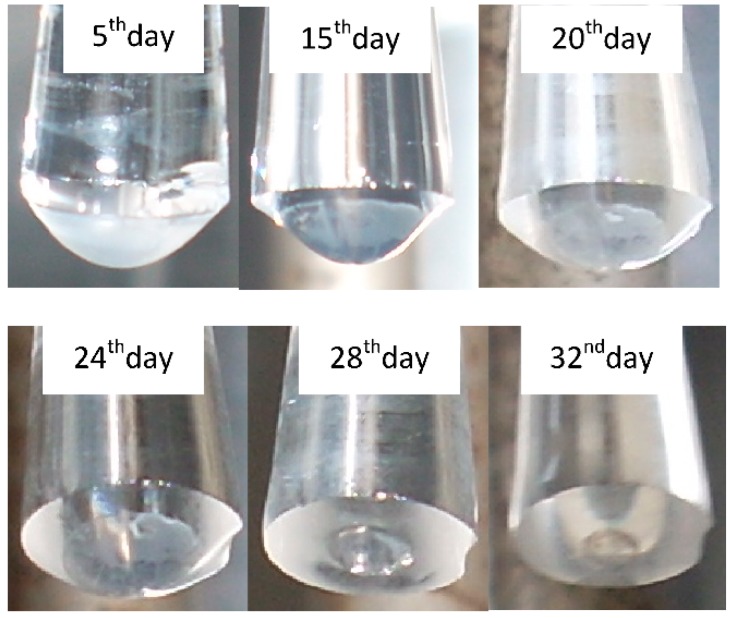
*Pseudomonas putida* TVA8 entrapped in silica gel on the end of quartz fiber tapered up. Changes during a one-month trial.

**Figure 18:**
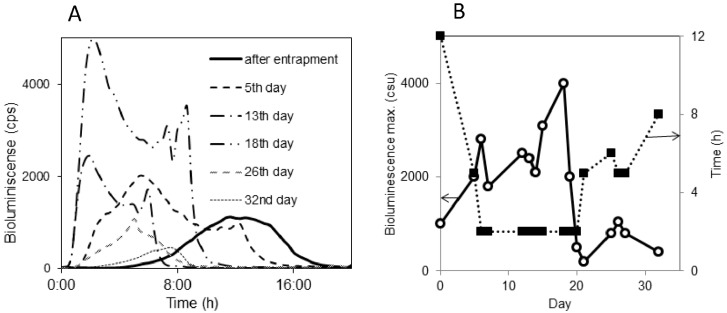
*Pseudomonas putida* TVA8 entrapped in a silica gel on the end of tapered up quartz fiber, during a one-month trial; (**A**) bioluminescence induced with toluene (26.5 g/L); (**B**) Bioluminescence maxima and response times of bioluminescence maxima.

With the aim of increasing the sensitivity of an optical fiber whole cell sensor of benzene, toluene, xylene and ethylbenzene (BTEX) bioluminescent bioreporter, *Pseudomonas putida* TVA8 were immobilized on the tip of tapered up quartz fiber (Figure 16f). The fiber tapering up makes it possible to increase the photon detection efficiency by increasing the number of the light sources, which are on the wider fiber end. The narrow end of the tapered optical fiber element was connected by a SubMiniature version A (SMA) optical fiber connector to a photon-counter. The bioluminescence of *Pseudomonas putida* TVA8, entrapped in prepolymerized tetramethoxysilane, was daily induced by immersion into a toluene solution (26.5 mg/L) in a phosphate saline buffer (pH = 7.2). The silica gel with entrapped cells was slowly dissolved over 32 days of trials (Figure 17). The first week, silica biogel covered the fiber end completely. With freshly entrapped cells, the maximum bioluminescence appeared after 12 h. In the following days, these response times became shorter due to biofilm formation on the surface of silica gel (Figure 18A). The minimum response times, 1–2 h, were observed between days 7 and 18. Later the response times were again prolonged and bioluminescence dropped down as the lens waned. Between the 7th and 18th days, the response times were 1–2 h and bioluminescence maxima from 2000 to 5000 cps (Figure 18B). At least within this period, the sensor worked as a reliable detector of toluene [191].

## 5. Immobilization of Biologicals for FOBS

**Figure 19:**
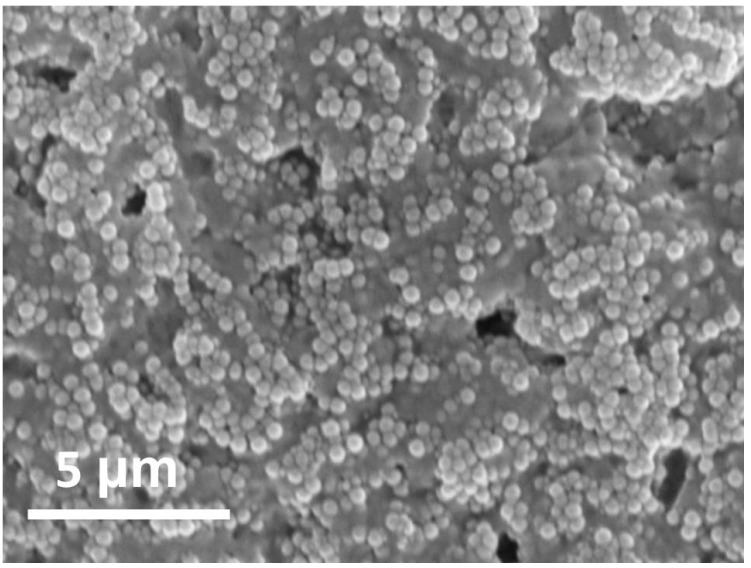
*Pseudomonas fluorescence* HK44 immobilized with silica particles Ø 306 nm.

The operational capability of devices with immobilized microorganisms are critically dependent on the ability to maintain immobilized bioreporter populations viably in a matrix that is strong enough to endure the rigours of the outside environment. The techniques of the immobilization of bioluminescent bioreporters that have been used or have a potential for application in the design of optical biosensors have been reviewed [156,171,194]. Their list comprises a broad spectrum of methods from bacterial biofilm in a flow-through microreactor [146], physical attachment enhanced by the modification of a substrate or live cell’s surface, well studied entrapment/encapsulation into natural or synthetic polymers, a combination of hydrogel entrapment and cryopreservation, plasma-deposited films, the application of photolithography or electrospinning and electrodeposition. In optical fiber sensors, bioreporters were fixed directly on the tips of OF or at the bottom of microtiter plate wells. To avoid irreversible analyte adsorption in polymer/gel matrices and a prolonged response time Premkumar *et al.* [195] embedded antibodies in a glutaraldehyde matrix and then attached *E. coli* bioreporter cells to the antibodies. Suspension of latex nanoparticles can be “painted” as thin nanoporous films onto solid substrates or used as ink to robotically print precise arrays of encapsulated cells [196]. Inorganic biofilm prepared from the silica nanoparticles (Figure 19) of the same dimensions as in latex biofilm had good cell viability but poor mechanical compactness. A comparative study of *Pseudomonas fluorescens 5RL* in the volume and surface of charged polyelectrolyte hydrogels and alginate gels demonstrated the ability of the hydrogels to immobilize the bioreporter without significantly affecting the physiology of the cells [197]. Immobilized and freely suspended cells *Pseudomonas putida* F1G4 (a bioluminescent biosensor of hydrophobic organic compounds) displayed similar bioluminescence profiles in response to hydrophobic compound exposure, but immobilized bacteria yielded a lower response level [198]. Mitchell and Gu studied 12 recombinant bioluminescent bacteria in liquid media and immobilized in agar and sol-gel matrices. Although the sensitivity of the immobilized cells was generally lower than cultures grown in liquid media, they were comparable. After 4 weeks, the majority of the strains used in both immobilized systems were still responsive [199].

### Immobilization in Silica Gel and New Concepts of the Immobilization of Living Cells and Enzymes for Implementation in Optical Devices

Since 1990, the sol-gel technology has been continuously explored to develop host materials for cell-based biotechnological devices. In biosensors design, the encapsulation of enzymes and living cells into inorganic or organic-inorganic matrices made by sol-gel process have been extensively applied. As host materials, silica gel and organic-inorganic polymers surpass organic polymers in chemical stability, biocompatibility, and a broad range of modifications of porosity, transparency and refractive index. The last two properties are advantageous especially for the OFS. Sol-gel immobilization techniques have been developed to minimize cells damage by extremes of pH, temperature and high concentrations of alcohol. The pH was adjusted close to pH = 7, alcohols preferably methanol, were evaporated before admixing the cells, or an aqueous route based on sodium silicate-silica nanoparticle mixtures were used to avoid cell membrane destruction by alcohol [200]. The cells were protected by nutrients [201] and additives such as glycerine [202], glycine, and betaine [203] or by pre-encapsulation into an alginate that have been used also in Al- or Zr-base biogels with *E. coli* [204]. Thin and homogenous silica coating on cells or these in alginate capsules was deposited from the vapor phase [205]. The effects of encapsulation into inorganic matrices on the physiology of microorganisms were reviewed [200] and there are many sol-gel immobilization reviews, such as [206,207,208], which showed many examples of sol-gel encapsulation in biosensor design [205]. Nevertheless, the dissolution of silica gel at pH = 7 undermines the firmness of the biorecognition component during a biosensor’s long term application or storage. As a consequence of these processes, a recognition silica-gel layer with cells on an optical fiber element slowly diminished (Figure 17) and active cells become situated preferably on the gel surface [191]. In the case of thin (1 mm) bacteria-silica layers, bacteria co-entrapped with a nutrient rich medium survived storage being immersed in a buffer for one year at 4 °C but the film was disintegrated. In humid ambiance at the same temperature, the compactness of the film and bacterial viability was sustained for 4 months [200].

The biocompatibility of silica precursors has been improved using organically-modified silica precursors. 3-aminopropyltriethoxysilane provides a better hold of biomacromolecules on the silica matrix through electrostatic interactions of their negatively charged groups with the silane amino group. This silane has gained wide acceptance for the immobilization of biomaterials.

The applications of modified silica precursors have diminished the negative influences of sol-gel encapsulation on enzymes. Silica precursors with an alkyl group that modified the hydrophobicity of a matrix improved the activity and stability of lipases [209]. The negative influence of alcohols evolving from three alkoxy groups and volume shrinkage of maturing silica gel (syneresis) has been suppressed when silica precursors were modified by exchanging alkoxy groups for polyols or glycerol. Water soluble tetrakis(2-hydroxyethyl)orthosilicate has been the base for the syntheses of hybrid nanomaterials with polysacharides, frequently used for encapsulating living cells such as alginate, κ-, τ- carrageenans and chitosan [210]. These organosilica approaches lead to a decrease in matrix pore size that can restrict the accessibility of the substrates and thus reduced viability of the entrapped cells [201]. Some technical limitations in the encapsulation of living cells into the recognition elements of biosensors by a sol-gel process might be overcome by reducing the contact time between the cells and sol-gel precursors [88] and incorporating phopholipids instead of surfactants [211,212].

A new direction for the encapsulation of biological entities into silica materials was started by the discovery of silicatein—the enzyme that catalyzes the formation of Si-O-Si siloxane bonds by means of a nucleophilic attack of the electronegative oxygen atom in their serine hydroxyl group at the electropositive silicon atom within the silicic acid molecule. As a result of the action of the catalytic center in the enzyme, the reaction proceeds even at silicic acid precursor concentrations below 1 mM in contrast to autopolycondensation of orthosilicic acid, in the absence of silicatein that only occurs at higher concentrations (>>1 mM) at neutral pH [213]. Silicatein-mediated biosilica immobilization of *E. coli* on the surface of SiO_2_ has already been demonstrated by the technique of Optical Waveguide Lightmode Spectroscopy [214]. Via *in vitro* biosilification reactions catalyzed with recombinant silicatein-α also optical waveguides have been prepared. The artificial biosilica fibers that mimic the natural sponge spicules had a refractive index of 1.47 (λ = 633 nm) and optical losses in the range of 5–10 dB·cm^−1^ [215]. A positive effect of biosilification encapsulation could be expected in enzymatic sensors. Through silaffin-mediated biosilicification of physically adsorbed *Candida antarctica* lipase B, a large improvement of enzyme thermostability was achieved [216].

Artificial spores are another novel concept of cell encapsulation based on bioinspired layer by layer (LbL) biosilification of individual cells [217]. Cells are encapsulated individually within thin and tough shells in a cytocompatible way, by mimicking the structure of bacterial endospores that survive under hostile conditions. The LbL coating process enables surface-functionalizability, which is useful in color identification and site-selective immobilization of encapsulated cells into optical devices. The LbL coating process has been studied for the deposition of nanomaterials such as magnetic nanoparticles that facilitated cell separation by a magnetic field and functional coating of optical fibers [218].

The natural process of the attachment of communities of microorganisms to a surface is biofilm formation. Tailored bioluminescent bioreporters biofilms grown on the recognition element of an optical biosensor is a further possible solution that will employ new findings of bacterial biofilm gene-expression patterns.

## 6. Conclusions/Outlook

The increasing commercial availability of various optical fibers and optical fibers elements such as tappers, FLPG, FBG and microrezonators opens the field for new designs and applications of FOS. The sensing principles of FOCS and FOBS are now focused mainly on fluorescence, SPR and interference techniques. The nanoscale size of currently available fluorescent nanoparticles permits measurements in an individual cell such as the concentrations of toxic chemicals in carcinoma cells. In contrast to other sensors, OF have features, which have allowed measurement in remote sites, which might be human organs, chemical reactors or the detection of environmental pollution, without electromagnetic interferences. It is exceptionally important for on-line monitoring in corrosive or explosive environment. The limits of FOS applications are determined by the material of the OF, which might be toxic and impurities decrease the sensor sensitivity, oftentimes ambient light affects the detected signal.

Currently, a comparison of oxygen and pH sensors shows best advantages and disadvantages of application of optical sensors instead of electrical ones. Among the most important advantages of optical oxygen sensors over traditional electrochemical sensors are; measurement of both gas and dissolved oxygen, temperature range from −80 °C to 80 °C and long life with less frequent calibration [219]. Optical sensors have the benefit that they do not consume the analyte (oxygen) and their measurement of dissolve oxygen is not dependent on mixing. Most commercially available optical oxygen sensors target the measuring range of 300 to 2 μmol·L^−1^. Optical Luminescence Measuring Oxygen Sensor (LUMOS) measures from 1000 nM down to the detection limit of 0.5 nM and exhibits lower noise, higher resolution and higher sensitivity than the electrochemical STOX sensor [220]. Various effects that could influence measurements with ultratrace optical oxygen sensors were described by Lehner *et al.* [221]. Imaging of a space distribution of concentrations as was demonstrated with pH planar optode [222] might be hardly realized with electrical sensors. High spatial resolution and measurement in very small sample volumes are domains of optical fiber pH meters, which in contrast to traditional ones have low drift and need not reference electrode [223]. Miniature optode on polymer optical fiber in blood serum reproducibly measured pH in the range pH = 3–9 with resolution 0.2 pH, for 24 h and response time 20 min. The simple fabrication from inexpensive materials made the optical sensor promising as a disposable device for monitoring wound healing instead of state-of-the art measurement with accurate but fragile glass microelectrodes or ion sensitive field effect transistors that suffer problems with separation of metallic components from fluid [224]. Commercially available optical pH sensors have range pH = 5–9 and at pH 7.0 resolution ± 0.01 pH units [225] in contrast to pH electrodes that might have range pH = −2–16 and resolution of pH ± 0.001 pH [226].

Time responses of optical chemical and biochemical sensors are frequently in minutes. Nevertheless bare optical fiber oxygen sensors have response times down <0.3 s [227], which overtakes typical time response of a commercial electrochemical sensor, 30 s [228] and fast galvanic electrode, 1 s [229]. The shortest time responses of optical pH sensors were >1 s [225]. In comparison with electrical sensors, the exploitation of the breadth and unexchangeable features of biological catalysts, enzymes and living cells, are still in an early stage. An assortment of optical fiber biosensors with oxidases should be dramatically enlarged only by the replacement of the oxygen electrode with an oxygen optrode. Similarly, only few techniques of enzyme immobilization have been applied in the preparation of optical fiber biorecognition elements.

The operational capability of devices with immobilized microorganisms is critically dependent on the ability to keep the immobilized cell populations viable and active. An encapsulation of biological entities in nanostructured matrices and a rational combination of immobilization techniques can provide a path toward the stable recognition element of an optical fiber biosensor. Effective immobilization might also overcome restrictions disabling wider utilization of GMO (especially bioluminescent or fluorescent bioreporters) as a part of the recognition elements of FOBS). Nevertheless we expect gradual liberation of the rules for GMO microorganisms handling hand in hand with increasing knowledge of their environmental spread and related risks.

## List of Symbols and Abbreviations

BCECF: 2',7'-bis-(2-carboxyethyl)-5-(and-6)-carboxyfluorescein
BOD: biological oxygen demand
BTEX: benzene, toluene ethylbenzeme and xylene
CCD: charge couple device
CTU: Czech Technical University
DNA: dinucleotide acid
EM: electro-magnetic field
EPA: U.S. Environmental Protection Agency
FBG: fiber with Bragg grating
FLPG: fiber with long period grating
FMNH_2_: reduced flavin mononucleotide
FMNH_2_: reduced flavin mononucleotide
FOBS: fiber optic biosensor
FOCS: fiber optic chemical sensor
FOE: fiber optic element
FOS: fiber optic sensor
GFP: green fluorescent protein
GI: gradient index
GMO: genetically modified organism
GO: geometry optics
GOX: glucose oxidase
HOPDA: chlorinated 2-hydroxy-6-oxo-6-phenylhexa-2,4-dienoic acid
HTPS: 8-hydroxypyrene-1,3,6-trisulfonic acid trisodium salt
IO: integrated optics
IR: infra-red spectral range
LbL: layer by layer
LOD: limit of detection
LUMOS: Luminescence Measuring Oxygen Sensor
MM: multi mode
NA: numeric aperture
NADH: nicotienamide dinucleotide reduced form
NADPH: nicotienamide dinucleotide phosphate reduced form
OF: optical fiber
PCB: polychlorinated biphenyls
PCS: polymer clad silica fiber
PMT: photomultiplier
POF: plastic optical fiber
RCHO: aliphatic aldehyde
RIU: refraction index unit
SEM: scanning electron microscopy
SI: step index
SM: single mode
SMA: SubMiniature version A
SPR: surface plasmon resonance
ssDNA: double-stranded DNA
ssDNA: single stranded DNA
UV: Ultraviolet spectral range
WGM: whispering galery mode
$\vec{E}$: vector of electric field intenzity
$\vec{B}$: vector of magnetic field
n(r): refractive index as function of diameter r
β: propagation constant
*P_0_*: light power in core
θ*_c_*: angle of skew ray with fiber axis
ε*_m_*: electric permitivity
μ*_m_*: magnetic permeabilty
γ_i_: modified absorption coeficient
*d_e_*: equivalent thickness
*R_c_*: critical bend radius of fiber
*M(R)*: reduced number of modes as function bend radius
*R_B_*: bend radius of fiber
*M*: number of guided modes
λ_c_: cut off wavelength
*E(x)*: evanescence field
*d_P_*: penetration depth
*α*(λ): attenuation on wavelength λ
ε: molar extinction coefficient
*C*: concentration
*Q*: quantum yield
*l*: optical path length in sample
*ψ_0_^max^*: maximal launching angle of light source
*r_max_*: maximal radius of light source
